# Assessing the origin of high-grade serous ovarian cancer using CRISPR-modification of mouse organoids

**DOI:** 10.1038/s41467-020-16432-0

**Published:** 2020-05-27

**Authors:** Kadi Lõhmussaar, Oded Kopper, Jeroen Korving, Harry Begthel, Celien P. H. Vreuls, Johan H. van Es, Hans Clevers

**Affiliations:** 1 Hubrecht Institute, Royal Netherlands Academy of Arts and Sciences and UMC Utrecht, Utrecht, The Netherlands; 2 Oncode Institute, Utrecht, The Netherlands; 3 Department of Pathology, UMC Utrecht, Utrecht, The Netherlands

**Keywords:** Genetic engineering, Cancer models, Ovarian cancer

## Abstract

High-grade serous ovarian cancer (HG-SOC)—often referred to as a “silent killer”—is the most lethal gynecological malignancy. The fallopian tube (murine oviduct) and ovarian surface epithelium (OSE) are considered the main candidate tissues of origin of this cancer. However, the relative contribution of each tissue to HG-SOC is not yet clear. Here, we establish organoid-based tumor progression models of HG-SOC from murine oviductal and OSE tissues. We use CRISPR-Cas9 genome editing to introduce mutations into genes commonly found mutated in HG-SOC, such as *Trp53*, *Brca1*, *Nf1* and *Pten*. Our results support the dual origin hypothesis of HG-SOC, as we demonstrate that both epithelia can give rise to ovarian tumors with high-grade pathology. However, the mutated oviductal organoids expand much faster in vitro and more readily form malignant tumors upon transplantation. Furthermore, in vitro drug testing reveals distinct lineage-dependent sensitivities to the common drugs used to treat HG-SOC in patients.

## Abstract (web summary)

The relative contribution of fallopian tube (FT) or ovarian surface epithelium (OSE) to high-grade serous ovarian cancer (HG-SOC) development is unclear. Here, the authors establish organoid models from murine oviductal and OSE tissues that allow cancer modeling via CRISPR-Cas9 genome editing, and report a dual origin of murine HG-SOC.

## Introduction

High-grade serous ovarian cancer (HG-SOC) is the most prevalent and aggressive gynecological malignancy that accounts for 70–80% of ovarian cancer mortalities^1^. Most ovarian cancer patients are diagnosed at a late stage, when the tumor has already metastasized throughout the abdominal cavity. As a result, the early stages of tumor development are not well characterized.

For years, it was believed that HG-SOC originates from the ovarian surface epithelium (OSE), which actively participates in the cyclical ovulatory rupture and repair processes^2^. It was assumed that the inflammatory environment induced by these processes exposed the OSE cells to oxidative stress and caused cell damage that could consequently lead to the accumulation of deleterious somatic mutations^3^. However, failure to identify HG-SOC precursor lesions in the OSE has led to the hypothesis that these carcinomas either arise de novo, without an intermediary lesion from epithelial inclusion cysts, or derive from an extra-ovarian source altogether.

Accumulating findings have shifted the focus away from the OSE toward the fimbria of the fallopian tube (FT). One of the first indications that suggested the FT as a possible origin of ovarian cancer were the lesions that were identified in the FT of high-risk patients carrying BRCA1/2 germline mutations^4–6^. These lesions, that are now referred to as serous tubal intraepithelial carcinomas (STICs), were found to carry mutations in the *TP53* gene, as present in almost all cases of HG-SOC (96%)^7,8^.

To study the potential of the OSE and FT to transform into HG-SOC, several models have been established. Studies with immortalized OSE cell lines as well as intrabursally administered viral particles that induce site-specific mutagenesis have been used to show the capability of OSE cells to generate different types of ovarian tumors in mice^9–13^. Mouse models that enable targeted mutagenesis in oviduct epithelium (the equivalent of human FT) via the use of tissue-specific gene promoters (such as *Pax8* or *Ovgp1*) have shown the ability of oviductal cells to transform into ovarian tumors^14–16^. In addition, several transcriptomic and proteomic analyses of human HG-SOC tissue support the dual origin of the cancer^17–22^. Yet, until now a direct comparison between the relative potential of oviduct and OSE to contribute to HG-SOC development has not been performed. Understanding the early stages of HG-SOC development and its tissue of origin is crucial for the design of early diagnosis and preventive strategies, especially for high-risk individuals such as women with *BRCA1* and *BRAC2* germline mutations.

Comparable HG-SOC models to study the tumor origin of OSE and FT in parallel have not been developed. In this study, we apply an organoid platform that enables a direct comparison of the two tissues of interest and, through in vitro engineering approach, elucidate their respective susceptibility to the disease.

## Results

### Derivation of organoids from murine oviduct and OSE

**Fig. 1:**
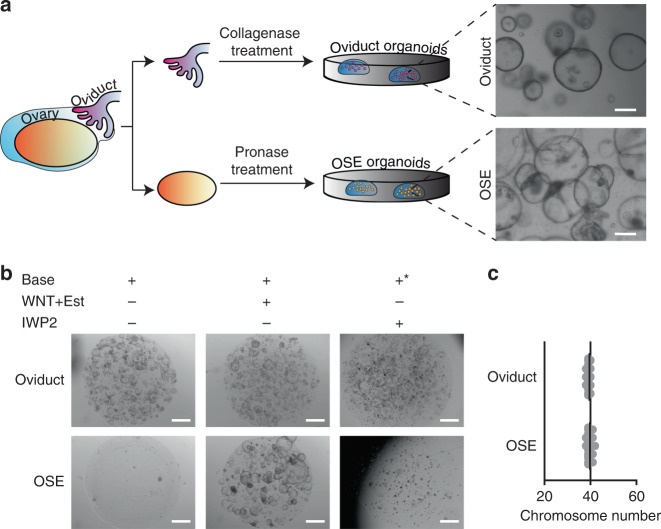
Derivation of organoids from murine oviduct and OSE. **a** For organoid derivation oviducts and ovaries were separated and subjected to collagenase or pronase treatment, respectively. The representative brightfield images of both organoid cultures are shown. Scale bar, 200 μm. The organoid derivation was reproducible over four independent experiments. **b** Basic medium requirement of oviductal and OSE organoids. OSE organoids are WNT-dependent. Representative images from *n* = 3 independent experiments. Est—Estrogen, IWP2—inhibitor of WNT protein 2, asterisk—in the WNT inhibitor conditions Rspo1 is withdrawn from the base medium. Scale bar, 2 mm. **c** Scatter plot presenting chromosome number distribution and mean of three independent lines, based on organoid metaphase spreads (20 spreads per line, three biological replicates, *n* = 60). Both wild-type organoid lines sustain healthy karyotype during culturing. **a**–**c** OSE—ovarian surface epithelium.

To derive long-term organoid cultures, mouse oviduct and OSE tissues were dissected, subjected to different enzymatic treatments, embedded in basement membrane extract (BME) and cultured in appropriate media (Fig. 1a and Supplementary Data 1). Using this protocol, oviduct and OSE cystic organoid formation was observed within 1–2 weeks following isolation (Fig. 1a).

It was previously shown that *Lgr5*, a key player in the WNT signaling pathway, marks stem cells of the murine OSE^23,24^, and that estrogen plays a stimulatory effect on OSE growth and proliferation^25^. In line with these observations, we found that OSE organoid growth is dependent on the addition of WNT protein to the medium, while oviduct organoids are not (Fig. 1b). The fact that oviductal cultures were not dependent on the addition of exogenous WNT suggests that these organoids either do not need active WNT signaling to grow or they produce their own WNT and, thus, are able to maintain growth through paracrine or autocrine WNT signaling. To rule out the latter, we exposed oviductal organoids to the IWP2 porcupine inhibitor which inhibits WNT O-acylation and secretion. IWP2 did not exert an inhibitory effect on oviductal organoid growth, confirming their WNT-independence (Fig. 1b). To avoid introducing additional variability to the analysis by culturing the lines in different media, oviductal and OSE organoids were both cultured in WNT- and estrogen-containing medium, unless stated otherwise. Oviductal and OSE organoid lines can both be expanded long-term (>1 year) while maintaining normal numbers of chromosomes, as demonstrated by metaphase spread analysis (Fig. 1c).

### Oviductal and OSE organoids show distinct characteristics

**Fig. 2:**
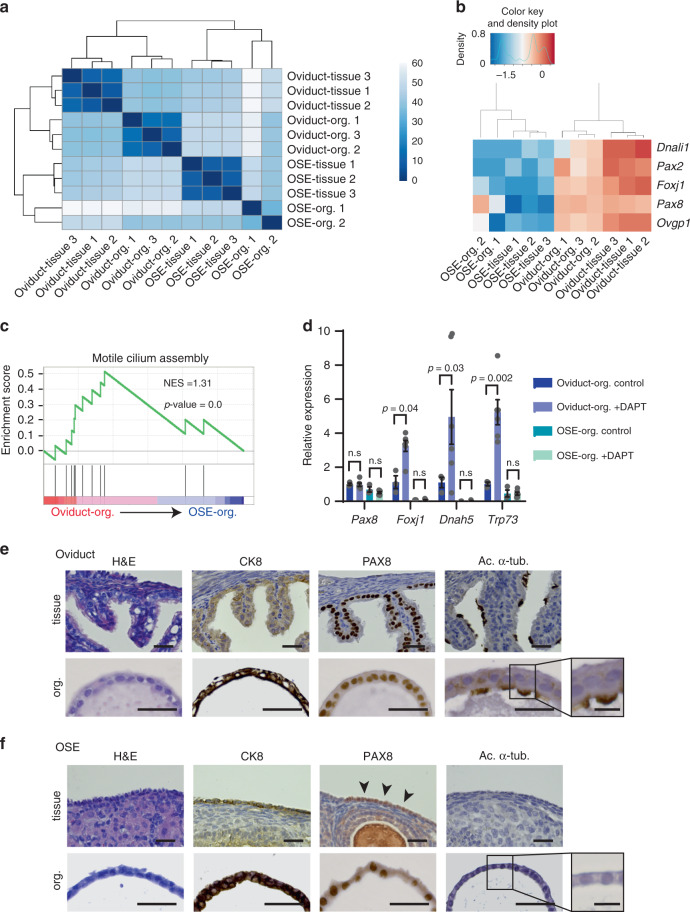
Characterization of healthy oviductal and OSE organoids. **a** Sample-to-sample heatmap showing the Euclidean distances between the organoids and parental bulk tissues as calculated from the regularized log transformation. Correlation is based on the top 500 differentially expressed genes between the organoid lines and the pseudocolor scale shows hierarchical distance from minimum (0, dark blue) to maximum (60, white). **b** Heatmap depicting known oviductal marker genes and their expression in the oviductal and OSE organoids and tissues. **c** Gene set enrichment analysis (GSEA) showing strong enrichment of genes involved in motile cilium assembly in oviductal organoids compared with OSE organoids. NES: normalized enrichment score, *p*-value is a permutation-based *p*-value that is computed and corrected for multiple testing (*n* = 3 oviductal and *n* = 2 OSE biologically independent organoid lines for each group). **d** qPCR results showing upregulation of selected genes (*Foxj1*, *Dnah5, Trp73*) involved in ciliogenesis in oviductal organoids upon treatment with NOTCH pathway inhibitor (DAPT) for 2 weeks. Assay was performed in *n* = 3 biologically independent replicates over three independent experiments. Error bars represent ±SEM. Statistical significance was calculated by two-tailed Student′s *t*-test, n.s—not significant. **e** Histological stainings of oviduct tissue and corresponding organoids (*n* = 4 independent experiments). The organoids are positive for epithelial marker CK8 and show presence of both PAX8-positive secretory and acetylated α-tubulin-positive ciliated cells. Scale bar, 25 μm; scale bar of the inset, 10 μm. **f** Histological stainings of OSE tissue and corresponding organoids (*n* = 4 independent experiments). OSE-derived organoids are positive for CK8 and PAX8, but lack ciliated cells. Scale bar, 25 μm; scale bar of the inset, 10 μm. **a**–**f** OSE—ovarian surface epithelium, Org.—organoids.

The endogenous oviduct is a monolayered epithelium that contains two main cell types, namely secretory and ciliated cells. In contrast, the OSE has no ciliated cells and is comprised of a monolayer of squamous-to-cuboidal epithelial cells^26^. Both oviductal and OSE organoids show cystic monolayered organoid growth, recapitulating the epithelium of tissues they were derived from (Fig. 1a, b). In order to compare the oviductal and OSE organoid lines more thoroughly, we performed RNA-sequencing (RNA-seq) analysis of parental tissues and organoids lines. Hierarchical clustering of the 500 most significantly differentially expressed genes between the oviductal and OSE organoid groups shows that oviductal organoids cluster together with the oviduct tissue and separate from the OSE organoids, which cluster with the OSE tissue (Fig. 2a). Importantly, our RNA-seq analysis allowed us to confirm the expression of several genes known to be specifically expressed in oviductal secretory (*Pax2*, *Pax8*, *Ovgp1*) and ciliated cells (*Dnali1*, *Foxj1*) in our oviductal organoids and tissues (Fig. 2b). These genes were largely absent in the OSE counterpart with the exception of oviductal secretory cell marker *Pax8* which was also found to be expressed in OSE organoids but not in the OSE tissue (Fig. 2b). Significant enrichment of motile cilium assembly genes in oviductal organoids was also confirmed by gene set enrichment analysis (GSEA) (Fig. 2c).

Previously, it has been established that *Pax8*-expressing secretory cells can serve as progenitor cells for ciliated cells and inhibition of NOTCH pathway has been shown to play a role in inducing ciliogenesis^27,28^. To assess the differentiation potential of the *Pax8*-expressing cells in our organoids, we treated both organoid lineages with the gamma-secretase complex inhibitor (DAPT). Consistent with their origin, oviductal organoids showed increased expression of ciliated cell markers upon NOTCH inhibition (Fig. 2d). Furthermore, the ciliated cells in oviductal organoids possessed beating cilia, confirming their functionality (Supplementary Video 1). In contrast, OSE organoids treated with DAPT did not form ciliated cells (Fig. 2d).

To characterize the oviductal and OSE organoids at the cell level, we performed immunohistochemical stainings for the epithelial marker, CK8, the secretory cell marker, PAX8, and the ciliated cell marker, acetylated α-tubulin (Fig. 2e, f). Both oviductal and OSE organoids were uniformly positive for CK8, confirming their epithelial origin (Fig. 2e, f). The presence of both secretory and ciliated cell types in oviductal organoids and tissues were confirmed by positive stainings for PAX8 and cilia (Fig. 2e). As revealed from RNA-seq analysis, OSE organoids also express the well-acknowledged oviductal secretory cell marker PAX8 (Fig. 2b, f). OSE tissue is largely PAX8-negative, however, rare PAX8-positive areas can be found in OSE lining and have been reported before (Fig. 2f, arrow heads)^29,30^. Altogether, characterization of oviductal and OSE organoids revealed distinct gene expression patterns that were consistent with their respective tissue of origin.

### Generating murine organoid models for HG-SOC development

**Fig. 3:**
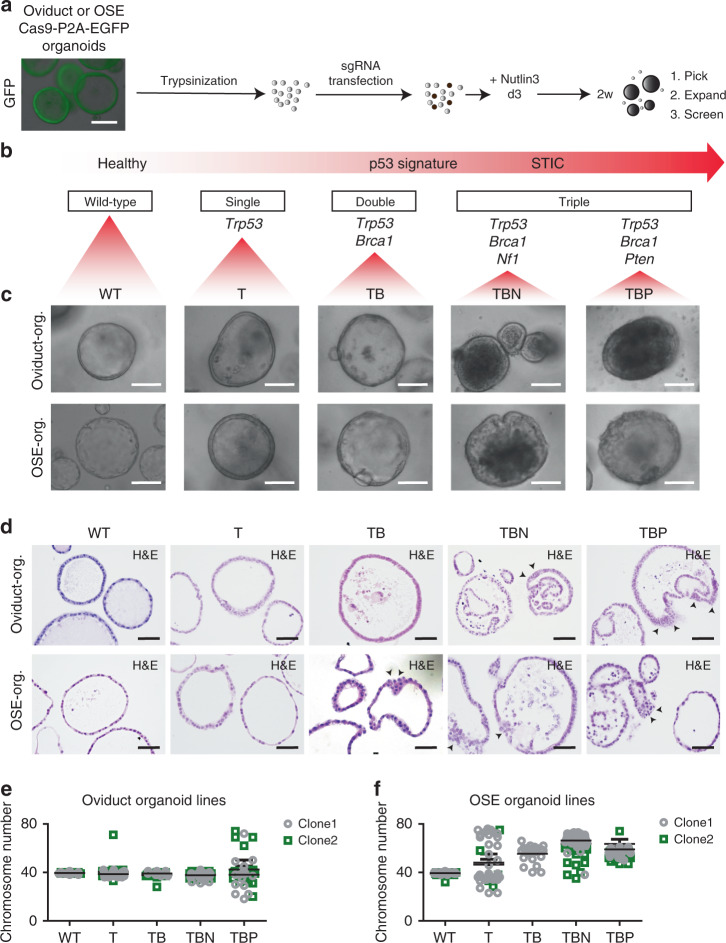
CRISPR-modification of oviductal and OSE organoids. **a** Strategy to generate the mutant lines using CRISPR-Cas9. The lines were trypsinized to single-cell suspension followed by sgRNA transfection. Three days after transfection Nutlin-3a was added to the medium to allow mutant *Trp53* organoid outgrowth. In 2 weeks emerging clonal organoids were picked, expanded and screened. Scale bar, 200 μm. **b** Hypothesized tumor progression model of HG-SOC and chosen genes to build comparable progression models. STIC—serous tubal intraepithelial carcinoma. **c** Brightfield images of comparable clones with the different set of mutations from both oviductal and OSE origin. Scale bar, 100 μm. **d** Immunohistochemical stainings for H&E to visualize the more detailed phenotype of oviductal and OSE clones. Arrow heads point to the cellular stratification. Scale bar, 50 μm. **c**, **d** Org.—organoids. **e**, **f** Scatter plots presenting chromosome number distribution and mean of **e** oviduct wild-type and mutant clones or **f** OSE wild-type and mutant clones, based on organoid metaphase spreads (*n* = 20 spreads per biologically independent clones). **c**–**f** OSE—ovarian surface epithelium, WT—wild-type, T—*Trp53* mutant; TB—*Trp53, Brca1* mutant; TBN—*Trp53, Brca1, Nf1* mutant; TBP—*Trp53, Brca1, Pten* mutant.

According to the current tubal HG-SOC development model^31^, mutations in the *TP53* gene, which are found in about 96% of cases, are considered an early event in tumor development, and can lead to “p53 signature” lesions, i.e., a linear stretch of cells that stain for mutant p53^31,32^. Upon accumulation of additional mutations, these lesions can gain proliferative capacity and generate serous tubal intraepithelial carcinomas^33,34^, also known as STICs, which then progress to invasive carcinoma. In addition to p53, the PI3K/RAS and homologous recombination (HR) pathways are commonly altered in HG-SOC (45% and 51% of cases, respectively)^7^. To establish in vitro oviduct and OSE tumor development models, we utilized CRISPR-Cas9 technology to target the murine *Trp53* gene alone or in combination with *Brca1*, *Pten*, and *Nf1* (Fig. 3a-b). These genes are recurrently mutated in human HG-SOC^7,35^.

We derived oviductal and OSE organoids from mice carrying a *Cas9-P2A-EGFP* expression cassette knocked into the ROSA26 locus, such that Cas9 and enhanced green fluorescent protein (EGFP) are expressed ubiquitously (Fig. 3a)^36^. Organoids were transfected with up to three different sgRNAs targeting the aforementioned genes, and then cultured for 2 weeks in the presence of Nutlin-3a to select for mutated *Trp53* cells^37^ (Fig. 3a). Surviving organoids were picked, clonally expanded, and their mutation composition was analyzed by targeted sequencing. We established clonal oviductal and OSE organoid lines containing single (*Trp53*; T), double (*Trp53*/*Brca1*; TB), and triple (*Trp53*/*Brca1*/*Pten*; TBP or *Trp53*/*Brca1*/*Nf1*; TBN) gene knockouts (Fig. 3b, c, Supplementary Fig. 1a-b). *Trp53* loss in single and double mutants was confirmed by western blot analysis (Supplementary Fig. 1c) and all the mutants were resistant toward Nutlin-3a treatment compared with the respective wild types (WT) (Supplementary Fig. 1d).

To achieve loss of function mutations, sgRNAs were designed to target promoter-proximal exons of the genes of interest (Supplementary Fig. 1a), and with the exception of *Brca1* sgRNAs, all the sgRNAs exhibited ≥50% targeting efficiency in organoids as calculated by the number of targeted clones that survived in Nutlin-3a selection (Supplementary Fig. 1a). *Brca1* sgRNAs showed a much lower efficiency, as only 15% of screened clones had been targeted by the sgRNA (Supplementary Fig. 1a). Not only was the *Brca1* gene difficult to target, but we could also never obtain clones in which both allels of *Brca1* carried out-of-frame mutations (i.e., completely knocked out), even on the background of *Trp53* mutation. Statistically, the observed targeting frequencies for *Brca1* locus do not fit with the expected frequencies as calculated based on our knowledge as to how the single allele is targeted (Supplementary Data 2). This suggests homozygous lethality of *Brca1*-null mutations in our rapidly dividing organoid systems. We performed a double-strand break (DSB) assay to test whether mutating a single allele of *Brca1* is sufficient to sensitize the cells to double-strand breaks. The heterozygous *Brca1-*mutants displayed significantly higher number of phosphorylated histone yH2A.X-positive cells upon treatment with the genotoxic agent Mitomycin C, confirming the presence of DSB repair defect in those clones (Supplementary Fig. 2a-c) and supporting haploinsufficiency of *Brca1* as previously reported^38^. We were successful in targeting all remaining genes of interest and created complete knockouts of these genes. Genetically modified organoids were derived from both oviductal and OSE origins with two independent clones for each combination of mutations. All the clones and their mutations are summarized in the tables under the Supplementary Fig. 1b.

Next, we characterized the lines more thoroughly by examining the differences in the histological, proliferative, and apoptotic properties of the clones. Single (T) and double (TB) mutant organoids from both origins did not show any apparent morphological change in culture, whereas the triple mutants (TBN and TBP) from both origins displayed a denser and folded appearance (Fig. 3c). This morphological difference was confirmed by hematoxylin and eosin (H&E) stainings where the triple mutants exhibited more irregular shapes and cellular stratification (Fig. 3d). In order to study the effect of different mutations on organoid growth speed, we closely monitored the expansion of the organoids by measuring the size of the organoids daily for a week (Supplementary Fig. 2d-e). In the oviductal lineage, we observed that all the mutation combinations induced significantly faster growth rate of the clones compared with the corresponding WT organoids (Supplementary Fig. 2d). In contrast, the growth rate of the OSE clones was largely unaffected upon introduction of mutations, with an exception of OSE-TBN and OSE-TBP lines that showed slightly higher or lower growth rate compared with the WT, respectively (Supplementary Fig. 2e). We also evaluated whether the levels of apoptosis differed between the lineages and the individual mutants. In oviduct-derived organoids, we observed a decrease in apoptosis upon introduction of mutations with the triple mutants possessing the least amount of apoptotic cells (Supplementary Fig. 2f). In contrast, in the OSE-derived organoids the effect appeared to be reversed as the triple mutants displayed more apoptotic cells than the single or double mutants (Supplementary Fig. 2f).

One of the hallmarks of HG-SOC is extensive chromosomal instability^7,8^. To evaluate the genomic stability of the mutant organoids, we performed metaphase spread analysis ~8 weeks after clonal selection of the single, double, and triple mutant clones (Fig. 3e, f). As expected, WT organoids from both origins displayed normal chromosome numbers (Fig. 3e, f). Aberrant chromosome numbers in genetically modified oviduct organoids were observed only in the TBP triple mutants (Fig. 3e), demonstrating the differential effect of the distinct mutated gene sets. Strikingly, in genetically modified OSE organoids, chromosomal abnormalities were already evident following a single knockout of the *Trp53* gene and were observed in double and triple mutant organoids as well, suggesting tissue-specific effects of *Trp53* mutation (Fig. 3f). Similarly to the OSE organoids, induction of extensive chromosomal abnormalities upon a single *Trp53* mutation has been observed before in human intestinal organoids^39^.

### Oviductal and OSE organoids show differential drug responses

**Fig. 4:**
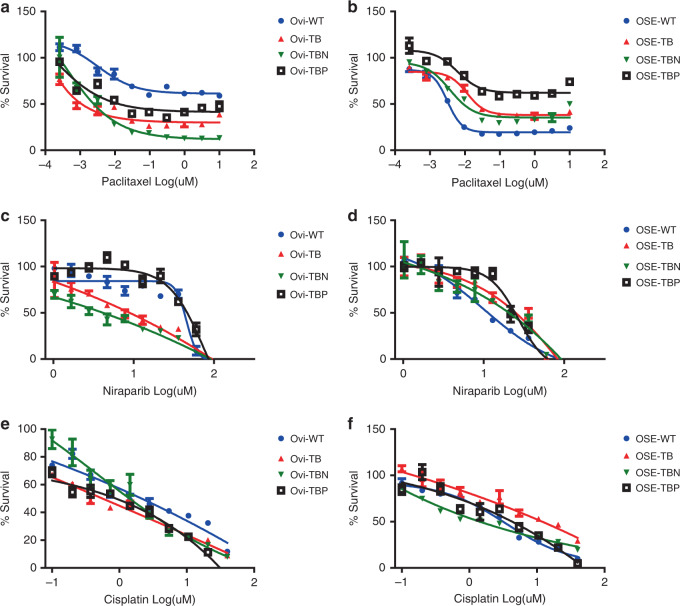
In vitro drug sensitivity of oviductal and OSE lines. **a**, **b** Representative dose–response curves of paclitaxel-treated **a** oviductal and **b** OSE clones. **c**, **d** Representative dose–response curves of niraparib-treated **c** oviductal and **d** OSE clones. **e**, **f** Representative dose–response curves of cisplatin treated **e** oviductal and **f** OSE clones. **a**–**f** Error bars represent ±SEM of quadruplicates (*n* = 4) over two independent experiments. Ovi—oviduct; OSE—ovarian surface epithelium; WT—wild type; TB—*Trp53, Brca1* mutant; TBN—*Trp53, Brca1, Nf1* mutant; TBP—*Trp53, Brca1, Pten* mutant.

We evaluated the drug sensitivity of our mutants to a set of chemotherapy regimens commonly used for HG-SOC treatment in patients, including cisplatin, paclitaxel, and niraparib (PARP-inhibitor). Both, paclitaxel and niraparib revealed differential sensitivities between the various mutant organoid sets derived from the two lineages (Fig. 4a–d). In the oviductal lineage, the lines were generally more sensitive to paclitaxel (Fig. 4a) and niraparib (Fig. 4c) upon acquiring more mutations. In contrast, in OSE lineage, the mutant lines were less sensitive to paclitaxel (Fig. 4b) and niraparib (Fig. 4d) compared with the WT line. As an exception, in the case of niraparib treatment, we noticed that the TBP triple mutants from both lineages behave differently from other mutants, showing the least sensitivity to the drug (Fig. 4c, d). It has been reported before that *Pten* mutation in the combination with *Brca1* mutation induces reversion in PARP-inhibitor sensitivity^40^. Our data support the finding. We did not observe distinct effects within mutants with the exposure to the common platinum-based drug, cisplatin (Fig. 4e, f).

Taken together, our data suggest that, dependent on their origin, the mutants show differential response to the two commonly used chemotherapeutic agents, which further substantiates the need to study the different sites of origin for HG-SOC more thoroughly.

### Tumorigenic potential of oviduct- and OSE-derived organoids

**Fig. 5:**
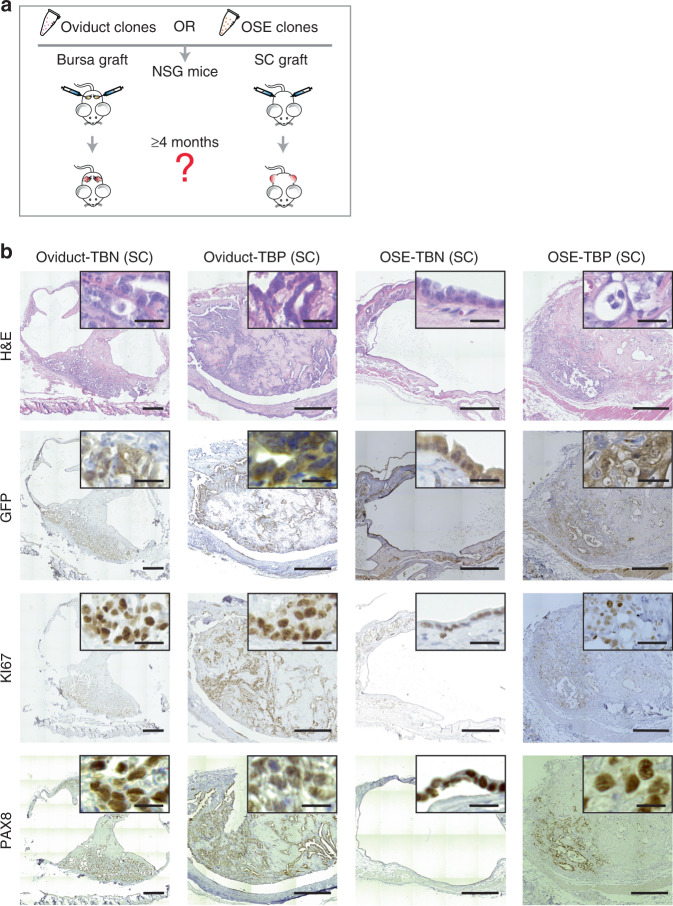
Both origins are able to give rise to HG-SOC-like tumors. **a** Transplantation strategy. The clones were transplanted either subcutaneously or orthotopically into the bursa of immunodeficient mice and tumor formation was assessed up to 4 months after organoid injections. Each mouse received two injections with the same clone into the opposite flanks or bursal cavities (*n* = 2 injections/mouse). **b** Representative histological overview and close-up images of subcutaneous tumors derived from transplantations with the different triple mutant oviductal (*n* = 12 tumors) and OSE (*n* = 7 tumors) clones. H&E, GFP, KI67, and PAX8 stainings are shown. Scale bar, 25 μm. OSE—ovarian surface epithelium; SC—subcutaneous; TBN—*Trp53, Brca1, Nf1* mutant; TBP—*Trp53, Brca1, Pten* mutant.

To assess their tumorigenic capacity, the genetically modified and WT organoids were orthotopically or subcutaneously transplanted into immunodeficient mice (Fig. 5a and Supplementary Data 3–4). As expected, none of the subcutaneously or orthotopically transplanted WT or *Trp53* single mutant organoids gave rise to tumors in either lineage (Table 1). In two cases (50% success rate, *n* = 4) tumor growth was observed following the subcutaneous oviduct-TB organoid transplantations, showing the potential of double mutants to acquire tumorigenic potential in vivo (Table 1). However, no tumor growth was observed with comparable OSE-TB organoids, irrespective of the transplantation method. Next, when genetically modified triple mutant organoids were orthotopically transplanted, both TBN and TBP oviduct triple mutant organoids formed tumors (92% success rate, *n* = 12 and 100% success rate, *n* = 12, respectively) (Table 1). In contrast, despite carrying the same oncogenic mutations, none of the orthotopically transplanted OSE organoids gave rise to tumors, including the TBN and TBP triple mutants (Table 1).

**Table 1:** Summary table of transplantation outcomes with oviductal and OSE organoids.

Origin	Clone	No. of injected cells	Orthotopic tumor take	Subcutaneous tumor take
Oviduct	WT	ca 100,000	0/6 (0%)	0/2 (0%)
OSE	WT	ca 100,000	0/6 (0%)	0/2 (0%)
Oviduct	T	ca 100,000	0/8 (0%)	0/4 (0%)
OSE	T	ca 100,000	0/8 (0%)	0/4 (0%)
Oviduct	TB	ca 100,000	0/8 (0%)	2/4 (50%)
OSE	TB	ca 100,000	0/6 (0%)	0/4 (0%)
Oviduct	TBN	ca 100,000	11/12 (92%)	6/6 (100%)
OSE	TBN	ca 100,000	0/8 (0%)	5/8 (63%)
Oviduct	TBP	ca 100,000	12/12 (100%)	6/6 (100%)
OSE	TBP	ca 100,000	0/8 (0%)	2/4 (50%)

OSE—ovarian surface epithelium, WT—wild type, T—*Trp53* mutant, TB*—Trp53, Brca1* mutant, TBN*—Trp53, Brca1, Nf1* mutant, TBP—*Trp53, Brca1, Pten* mutant.

When the genetically modified organoids were subcutaneously transplanted, both TBN and TBP oviduct triple mutant organoids developed into tumors (100% success rate, *n* = 6 and 100% success rate, *n* = 6, respectively) (Table 1). Surprisingly, although TBN and TBP OSE-derived triple mutants were not able to form tumors following orthotopic transplantations, they did form tumors after subcutaneous transplantions with 63% (*n* = 8) and 50% (*n* = 4) success rate, respectively (Table 1). Taken together, these results indicate that both oviductal epithelium as well as OSE can give rise to ovarian tumors. However, the inability for the OSE lineage to do so at the orthotopic site in the given time frame (4 months) suggests that tumors from OSE arise with slower kinetics.

Further analysis of the mice that were injected with WT, single (T), double (TB), or triple (TBN/TBP) mutant organoids that did not develop tumors revealed minute non-proliferative cystic remnants of the tranplantations (Supplementary Fig. 3a), confirming the success of the organoid transplantations and highlighting their benign nature. Taken together, although both oviduct and OSE organoids can give rise to ovarian tumors, oviduct-derived organoids appear to hold considerably higher tumorigenic potential.

Next, tumors were isolated and their histological properties were analyzed. H&E staining analysis revealed two distinct phenotypes: cystic (*n* = 18) and solid tumors (*n* = 26) (Fig. 5b, Supplementary Fig. 3b-c, Supplementary Data 3–4). These phenotypes had no apparent correlation with the tissue of origin nor transplantation site. To distinguish organoid-derived tumor cells from the recipient normal mouse cells, tumors were stained with an anti-GFP antibody which confirmed that the tumors originated from the transplanted clones (Fig. 5b, Supplementary Fig. 3b-c). Interestingly, in a subset of solid tumors this staining revealed a population of GFP-positive cells with mesenchymal features that surrounded glandular tumor structures (Supplementary Fig. 3b), indicating the occurrence of an epithelial-to-mesenchymal transition (EMT).

All tumors from either origin ubiquitously stained for PAX8 (Fig. 5b)—a well-known marker for HG-SOC, with the exception of solid tumors that consisted of both glandular and mesenchymal-like tumor cells (Supplementary Fig. 3b). In these tumors only the glandular structures were positively stained for PAX8, and were surrounded by PAX8-negative mesenchymal-like cells, suggestive of involvement of EMT in *Pax8* downregulation (Supplementary Fig. 3b). Mesenchymal features and EMT are often associated with tumor metastasis. Indeed, all oviduct-derived orhotopic tumors displayed abdominal wall metastases (Supplementary Fig. 3d).

Oviduct-derived tumors were generally more proliferative as shown by bigger tumor sizes (Supplementary Fig. 3e) and higher KI67 labeling index (Supplementary Fig. 3f, Fig. 5b). As a result of the more proliferative tumor growth, the oviductal tumors also displayed significantly higher number of apoptotic cells as quantified from the cleaved Caspase-3 stainings in the tumors (Supplementary Fig. 3g). As determined by a certified pathologist, solid tumors from both oviductal and OSE origins displayed histologically human HG-SOC-like features, including glandular growth and serous papillary structures. However, the cystic tumor histology we observed was atypical to HG-SOCs (Fig. 5b, Supplementary Fig. 4a). Nevertheless, the cystic tumors showed no mucinous architecture or presence of conspicuous cilia, excluding the diagnosis of benign mucinous borderline tumor or serous borderline cystadenoma, respectively, and displayed malignant features such as abundant mitotic figures, pleomorphism and nuclear atypia suggestive of malignant type of ovarian carcinoma (Fig. 5b, Supplementary Fig. 4a-c).

### Oviduct tumor-derived organoids recapitulate tumor evolution

**Fig. 6:**
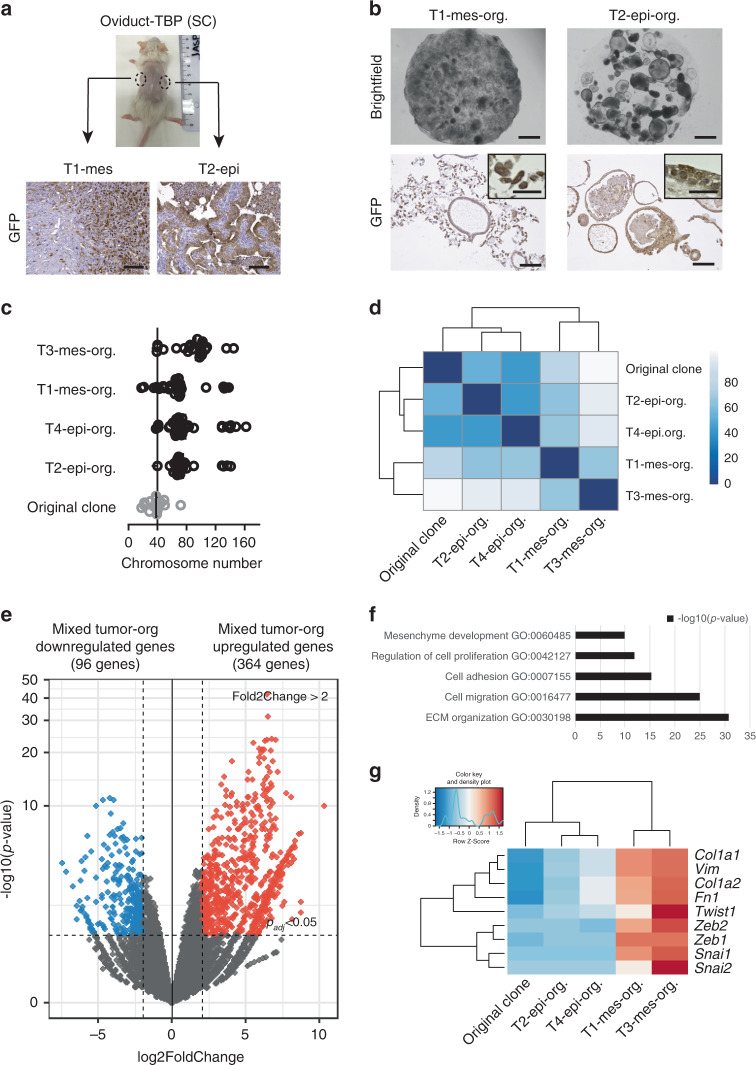
Oviduct tumor-derived organoids recapitulate tumor evolution. **a** Representative image of a mouse showing left and right side subcutaneous tumors derived from oviduct-TBP clone injection. Distinct histological phenotype of two adjacent subcutaneous tumors (labeled as T1-mes and T2-epi according to the histological phenotype) injected with the same oviduct-TBP clone. GFP stainings are shown (*n* = 4 tumors observed). Scale bar, 100 μm. **b** Brightfield images (top) as well as GFP stainings (bottom) of T1-mes and T2-epi tumor-derived organoid cultures (labeled as T1-mes-org. and T2-epi-org., org.—organoids). Brightfield image scale bar, 2 mm. GFP-stained image scale bar, 100 μm; inset scale bar, 25 μm). **c** Scatter plot presenting chromosome number distribution and mean of oviduct-TBP original clone and resulting four independent tumor-derived organoid lines (labeled as T1-mes-org., T2-epi-org., T3-mes-org., and T4-epi-org., org.—organoids), *n* = 20 spreads per biologically independent clone. **d** Sample-to-sample heatmap showing the Euclidean distances between the original oviduct-TBP clone and the resulting tumor-derived organoids as calculated from the regularized log transformation. Correlation is based on all the differentially expressed genes in the dataset and the pseudocolor scale shows hierarchical distance from minimum (0, dark blue) to maximum (100, white). **e** Volcano plot of RNA expression comparison between the organoids derived from epithelial (*n* = 2) and epithelial-mesenchymal tumors (*n* = 2). Red: significantly upregulated genes (log2FoldChange > 2, *p*_adj_ < 0.05) in organoids with mixed epithelial-mesenchymal phenotype. Blue: significantly downregulated genes (log2FoldChange < −2, *p*_adj_ < 0.05) in organoids with mixed epithelial-mesenchymal phenotype. **f** Significantly enriched biological processes in mixed epithelial-mesenchymal cultures. **g** Heatmap showing upregulation of selected EMT-related genes in organoids that were derived from tumors displaying mesencymal phenotype.

As described previously, we occasionally observed that the oviductal solid tumors displayed two distinct morphologies: glandular epithelial (epi) or mixed epithelial-mesenchymal-like (mes) phenotype. However, the tumor morphology was not a clone-dependent feature as the same triple mutant oviductal clone could give rise to both histopathological appearances at adjacent locations. As an example, we injected oviduct-TBP mutant organoids subcutaneously into the left and right flanks of the immunodeficient mice (Fig. 6a). Visible tumors grew on both sides of the mice and subsequent histopathological examination revealed that some mice developed tumors that displayed distinct morphologies at adjacent subcutaneous injection sites (Fig. 6a).

To determine whether these morphological differences could be recapitulated in the culture system, we derived independent organoid lines from four distinct-looking oviductal tumors (2 epi- and 2 mes-like tumors). To specifically select for the growth of the tumor-derived cells, organoids were derived and grown in Nutlin-3a supplemented medium (Fig. 6b). Consistent with the differential tumor histotypes we observed in vivo, we found remarkable differences between the organoids derived from these tumors (Fig. 6b). The mesenchymal-like tumors gave rise to the mixed-like organoid cultures (T1-mes-org. and T3-mes-org.) that contained both cystic epithelial organoids as well as fibroblast-like cells (Fig. 6b). All cells expressed GFP, confirming that they were derived from the same original oviductal TBP clone (Fig. 6b). In contrast, the organoid cultures (T2-epi-org. and T4-epi-org.) derived from the glandular subcutaneous tumors resulted in the GFP-positive organoids that were uniformly epithelial and had no detectable fibroblast-like component, recapitulating the parental tumors (Fig. 6b). Subsequent metaphase spread analysis revealed that all the four tumor-derived organoid lines had acquired more widespread chromosomal abnormalities compared with the original clone, likely reflecting the tumor cell evolution in vivo (Fig. 6c).

Next, we analyzed gene expression patterns of these four independent organoid lines that were derived from mesenchymal- and epithelial-like tumors (Fig. 6d). Gene expression analysis revealed that the cultures that displayed uniform epithelial phenotype (T2-epi-org. and T4-epi-org.) clustered together with the original oviductal TBP clone, whereas the mixed tumor-derived cultures (T1-mes-org. and T3-mes-org.) formed a separate group (Fig. 6d).

To determine biological pathways and patterns enriched in the mixed-type tumor-derived organoids, we performed comparison between the organoids derived from the distinct types of tumors (Fig. 6e). Among the 364 genes upregulated in the mixed-type organoids (Fig. 6e), there was a significant enrichment for the biological processes related to the extracellular matrix (ECM) organization, cell migration, cell adhesion, cell proliferation, and mesenchyme development (Fig. 6f). More detailed gene expression analysis revealed that, in compliance with the epithelial-mesenchymal phenotype, the mixed-type organoid cultures showed upregulation of several EMT-related genes (e.g., *Vim*, *Twist1*, and *Zeb1/2*) compared with the original clone, while non-mesenchymal tumor derivatives largely lacked the expression of such genes (Fig. 6g).

### Oviductal tumors resemble the molecular subtypes of HG-SOC

**Fig. 7:**
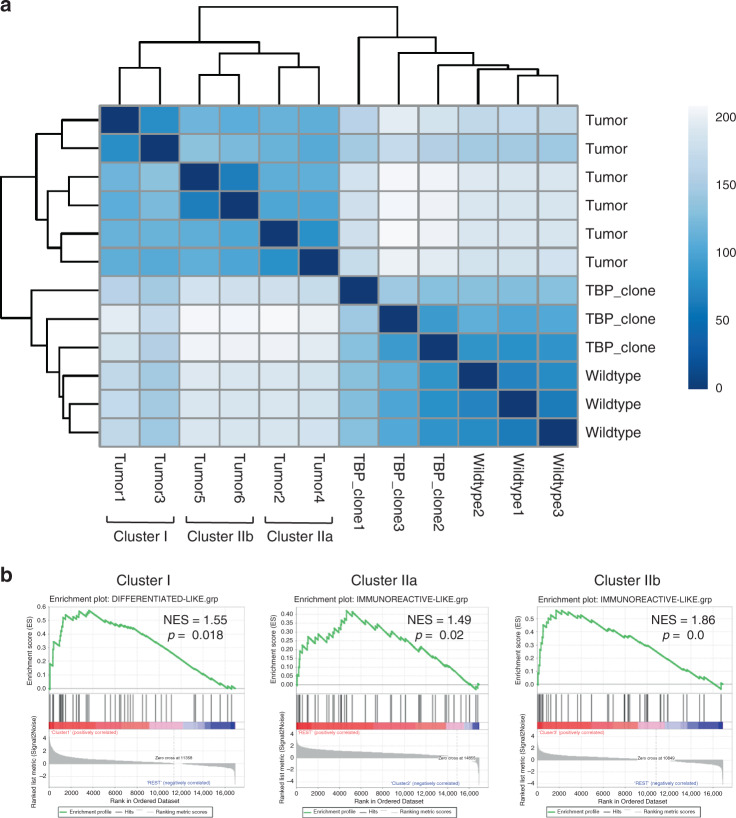
Oviductal tumors resemble the molecular subtypes of HG-SOC. **a** Sample-to-sample heatmap showing the Euclidean distances between the oviductal wild-type organoids, TBP-clones and six independent TBP-clone-derived tumor tissues as calculated from the regularized log transformation. Correlation is based on all the differentially expressed genes between the samples and the pseudocolor scale shows hierarchical distance from minimum (0, dark blue) to maximum (200, white). Clustering assigns the tumors into three distinct clusters (Cluster I, IIa, and IIb). TBP—*Trp53, Brca1, Pten* mutant. **b** GSEA showing strong enrichment for genes characteristic to human differentiated- and immunoreactive-like HG-SOCs in different tumor clusters. NES: normalized enrichment score, *p*-value is a permutation-based *p*-value that is computed and corrected for multiple testing.

Four molecular subtypes of human HG-SOC—mesenchymal, immunoreactive, differentiated, and proliferative—have been previously identified^7,41^. We therefore sought to analyze to what extent our tumors resembled human molecular subtypes. As the majority of the OSE-derived tumors were too small for saving a material for anything beyond histological analysis, we performed RNA-sequencing on 6 of the oviductal TBP clone-derived tumors (labeled as Tumor 1–6) to assess whether these tumors, originating from the same genetic background (i.e., TBP mutants), show a resemblance to any of the previously identified molecular subtypes of HG-SOC. Hierarchical clustering analysis including oviductal WT organoids, TBP mutants, and the six independent oviductal TBP clone-derived tumors assigned the organoids and the tumor tissues into two separate clusters (Fig. 7a). Interestingly, the six tumors mapped to a single branch, but were then further divided into two main clusters (clusters I and II) with the cluster II splitting into two additional subclusters (IIa and IIb) (Fig. 7a). Based on this clustering, we performed gene set enrichment analysis with the known signature genes upregulated in all the four human molecular subtypes previously identified^42^. The analysis revealed that while the tumors in cluster I had significant enrichment for the differentiated-like subtype (NES = 1.11, *p* = 0.018), the tumors in clusters IIa and IIb were most similar to the immunoreactive-like subset of HG-SOCs (NES = 1.49, *p* = 0.02 and NES = 1.86, *p* = 0.0, respectively) (Fig. 7b). Taken together, this data suggest that murine oviduct organoid-derived tumors are able to give rise to a varied set of human HG-SOC-like tumors as shown by their resemblance to different molecular subtypes.

## Discussion

In this study, we employ organoid technology to study the origin of HG-SOC. The current consensus in the field states that the fallopian tube is the main origin for HG-SOC. However, accumulating evidence indicates that ovarian surface epithelium might also give rise to a smaller subset of tumors^17–22^. To document the possible relative contributions of both hypothesized origins to HG-SOC, we have established 3D murine organoid cultures from both lineages to model the disease. Organoids derived from oviduct and OSE show evident differences in their gene expression. Surprisingly, OSE organoids express *Pax8*, a well-known oviductal secretory cell marker commonly used for the diagnosis of HG-SOCs. This finding suggests that *Pax8* might not serve as straightforward marker for oviduct-derived HG-SOCs as previously thought. Instead, both tissues as well as their organoid-derivatives can express *Pax8*.

Previously, it has been shown that organoid cultures can serve as reliable systems for studying tissues in health^28,43,44^ and disease^45–47^, and for modeling cancer development^39,48–51^. During revision of our paper, a parallel study was published where organoids were used to investigate HG-SOC. However, the introduced mutations and targeting constructs were limited to only two genes, *Rb* and *Trp53*^52^. Here, we introduced common HG-SOC mutations into oviductal and OSE organoid cultures, including *Trp53*, *Brca1*, *Nf1*, and *Pten*. Surprisingly, we could never obtain a homozygous *Brca1* knockout in our organoids, yet all the relevant *Brca1* heterozygous mutants displayed deficiency in the homologous recombination pathway. Homozygous knockout of *Brca1* is embryonically lethal^53,54^, yet possible in tissue-targeted transgenic models^15^. However, the number of targeted cells in vivo is much higher compared with our in vitro engineering approach which might support the survival of homozygous mutations. Alternatively, the in vitro growth medium environment might not support genomically destabilizing mutations such as those in the *Brca1* gene. The mutant clones from the two lineages showed differential genomic stability as well as differential changes in proliferation and apoptosis upon acquiring more mutations. In addition, distinct lineage-specific response was observed with two common HG-SOC drugs, paclitaxel and niraparib, substantiating the importance of studying the possible dual origin of human HG-SOC.

The contribution of the mutant clones derived from either of the two origins to tumor development was assessed by subcutaneous and orthotopic transplantation assays. Our results demonstrate that both tissues are able to give rise to tumors that recapitulate the histopathology of human HG-SOCs. However, oviductal tumors outperform the OSE tumors by several aspects, such as more successful tumor-derivation and higher proliferation rate. Interestingly, OSE-derived clones were not able to grow as orthotopic tumors, suggesting the presence of regulatory cues that specifically inhibit the survival or proliferation of the OSE, but not the oviductal derivatives, in the bursal environment, despite the similar mutation background. In addition, our data suggest that the tumors arising from murine oviductal origin are able to genetically resemble distinct molecular subtypes previously identified in human HG-SOCs^7,41^.

In conclusion, our model supports the prevalent standpoint in the field that the majority of HG-SOCs arise from the fallopian tube. Albeit, to a lesser extent, OSE is also capable of giving rise to HG-SOC-like tumors. Further research may substantiate these results in a human organoid setting, help identifying novel biomarkers to distinguish OSE- from FT-derived HG-SOCs, and uncover the possible differential clinical behaviors of the tumors from these two origins.

## Methods

### Mice

Wild-type *C57BL/6* and *B6J.129(B6N)-Gt(ROSA)26Sor*^tm1(CAG-cas9*,-EGFP)Fezh/J^ (JAX stock #026175)^36^ mice strains were used for derivation of wild-type and *Cas9/GFP*-expressing organoids, respectively. For mutant organoid transplantations, NOD scid gamma (*NSG; NOD.Cg-Prkdc*^*scid*^ *Il2rg*^*tm1Wjl*^*/SzJ* mice were used. Transplantation experiments were performed after institutional review by the Animal Ethics Committee of the Royal Netherlands Academy of Arts and Sciences (KNAW) with project license of AVD8010020151 and research protocol HI17.1001.

### Human specimens

The patient tumor material was collected from consenting patients undergoing surgical resection at the University Medical Center (UMC) Utrecht Hospital. Tissue collection was approved by the medical ethical committee UMC Utrecht under the biobanking protocol: 14-472 HUB-OVI. All patients participating in this study signed the informed consent forms.

### Derivation of oviductal organoids

For organoid derivation, ovaries and oviducts were dissected from mice and carefully separated under the stereo microscope. Oviducts were first placed into a collagenase solution (1 mg/mL of collagenase from *Clostridium histolyticum*, Sigma, C9407) in AdDF+++ (advanced DMEM/F12 supplemented with 1x Glutamax, 10 mM HEPES and penicillin-streptomycin, all from Invitrogen) with ROCK inhibitor (Y-27632, 10 µM). The tissue was incubated at 37 °C for 2 h while shaking followed by vigorous mechanical sharing using a fire-polished glass pipette and centrifugation for 5 min at 450 × *g*. The material was further digested with TrypLE (Gibco, Cat. 12605-010) for 5 min at 37 °C, washed several times with ice-cold AdDF+++ and embedded in Basement Membrane Extract (Cultrex^®^ BME RGF type 2, Amsbio, Cat. 3533-005-02). The cell-BME suspension was plated on a pre-warmed suspension culture plate (Greiner) and allowed to solidify at 37 °C for 30 min before addition of medium. The basal culture medium for oviductal organoids includes 20% R-spondin conditioned medium (made in-house), 1% Noggin conditioned medium (U Protein Express), 1x B27 (Gibco), 1.25 mM n-Acetylcystein (Sigma), 50 ng/ml EGF (Peprotech), and 500 nM A83-01 (Tocris). WNT inhibitor IWP2 (3 μM, Stemgent) was used for showing WNT-independency of oviductal organoids. For induction of ciliogenesis, DAPT (10 µM, Tocris) was added to the WNT-deprived culture medium and analysis was performed 2 weeks after the start of the treatment. The organoids could be reproducibly derived from over four independent isolations.

### Derivation of OSE organoids

Ovaries were subjected to more gentle treatment with pronase solution (1 mg/ml of Pronase E, Sigma) in AdDF+++ with ROCK inhibitor at 37 °C for 30 min while shaking. This method allows removal of OSE cells from the surface of the ovaries while leaving rest of the tissue intact. After digestion the ovaries were gently sheared a few hundred times by using an adjusted 1-ml pipette tip (tip hole needs to be cut large enough for the ovaries to enter without breaking them) to detach the loosened OSE sheets from the surface of the ovaries. Next, the supernatant containing epithelial fragments was transferred to another tube, pelleted by centrifugation for 5 min at 450 × *g* and further digested with TrypLE for 5 min at 37 °C. After several washes with ice-cold AdDF+++, the cells were embedded in BME and plated on pre-warmed culture plates as described above. The minimal required medium for OSE organoids contains all the factors present in the oviductal organoid medium (see above), but is additionally supplemented with 50% conditioned WNT3a (made in-house) and 0.1 μM β-Estradiol (Sigma). Addition of FGF2 during initial passages can improve the organoid outgrowth. WNT inhibitor IWP2 was used for demonstrating WNT-dependency of OSE organoids. For ciliogenesis assay, cultures were grown in the presence of DAPT for 2 weeks until analysis. The organoids could be reproducibly derived from over four independent isolations.

### Organoid growth assay

Organoids were removed from BME and trypsinized with TrypLE. Cells were washed in medium and passed through a 40 μm cell strainer to ensure a single-cell suspension. Cells were diluted in trypan blue to exclude dead cells and counted using a haemocytometer. One-thousand cells were plated with 5-μl BME drops into 48-well plates (Greiner Bio-One, 677102) and overlaid with 250-μl medium. Images of the organoids were taken every day for a week, organoid sizes were measured (12 organoids per line) using ImageJ software (version 1.51j8). Subsequently, from the resulting data, the growth curves were constructed in Microsoft Excel 2019. Experiment was repeated twice.

### Flow cytometry

To assess the apoptosis in organoids, the cells were stained and analyzed by flow cytometry. Organoids were collected and dissociated into single-cell suspension via trypsinization. The cells were stained with Annexin V Apoptosis Detection Kit (88-8007-72, eBioscience) according to the manufacturer′s instructions. A BD FACSCanto II system was used to analyze the samples. The assay was performed twice. The gating strategy is provided in the Supplementary Fig. 5.

### Immunohistochemistry

Tissues were fixed overnight in 4% paraformaldehyde at 4 °C followed by dehydration and paraffin embedding. To prepare organoids for histological stainings, intact BME drops containing organoids were collected from the culture plates and incubated in Cell Recovery Solution (Corning, Cat. 354253) on ice for 30 min, occasionally inverting the tube, to dissolve BME. Organoids were then allowed to settle to the bottom of the tube by free gravitation, supernatant removed and the material fixed in 4% paraformaldehyde at room temperature for 1 h. After fixation, the organoids were washed in PBS, and embedded into paraffin blocks. Sections were cut and hydrated before staining. Sections were subjected to H&E staining or immunohistochemistry by using antibodies listed in the Table 2. The images were acquired on Leica DM4000 microscope and processed using Leica LAS X software.

**Table 2:** Antibody specifications.

Antibody	Company, Cat#	Dilution	Incubation	Antibody retrieval
PAX8	Proteintech, 10336-1-AP	1:2000	Overnight, RT	Citrate buffer, pH 6.0
Ac-alpha-Tubulin	Santa Cruz, sc-23950	1:2000	Overnight, RT	Citrate buffer, pH 6.0
KI67	Monosan, MONX10283	1:2000	Overnight, RT	Citrate buffer, pH 6.0
Cytokeratin-8	Santa Cruz, sc-101459	1:50	Overnight, RT	Citrate buffer, pH 6.0
GFP	Life Technologies, A11122	1:1000	Overnight, RT	Citrate buffer, pH 6.0
Cleaved Caspase-3 (D175)	Cell Signaling Technology, #9661L	1:500	Overnight, RT	Citrate buffer, pH 6.0

### Double-strand break repair assay

Organoids were treated overnight with 15 µM Mitomycin C (Sigma, M4287). About 16 h later, organoids were harvested and fixed in 4% formalin overnight at 4 °C. Prior to the whole-mount staining, the fixed organoids were permeabilized with 0.5% Triton-X (Sigma), 2% donkey serum (BioRad) in PBS for 30 min at 4 °C and blocked with 0.1% Tween-20 (Sigma) and 2% donkey serum in PBS for 15 min at room temperature. Subsequently, organoids were stained with mouse anti-γH2A.X primary antibody (1:500, Millipore, clone JBW301) overnight at 4 °C, followed by four washes with PBS and incubation with secondary goat anti-mouse AF-647 antibody (1:250, Thermo Fisher, catalog number A-21235) for 2 h at room temperature in the dark and washed again with PBS. DAPI was used to counterstain nuclei. Organoids were mounted and imaged on an SP8 confocal microscope (Leica). Fluorescent microscopic images of γH2A.X were quantified as follows: Based on the staining, the nuclei were classified as γH2A.X-positive or -negative. The fraction of positively stained nuclei over all nuclei is displayed as one datapoint per organoid. At least 10 organoids were quantified per line over two independent experiments.

### Karyotyping

About 2–3 days after splitting of the organoids, the cultures were treated with 0.1 μg/mL colcemid (Gibco, Cat. 15210-040) added to the culture media for 6 h. Organoids were then collected and dissociated into single cells using TrypLE. Single cells were swollen by addition of pre-warmed 75 mM KCl and incubated at 37 °C for 10 min. Cells were fixed by slow drop-wise addition of ice-cold methanol:acetic acid (3:1) while gently tapping the cell suspension. Slides were mounted with DAPI-containing Vectashield, imaged on a DM6000 Leica microscope with a ×100 objective, and quantified by manual chromosome counting. At least 15 spreads per clone were analyzed.

### Organoid transfection and genotyping

For generating mutant organoid lines for selected genes (*Trp53*, *Brca1*, *Nf1*, and *Pten*), the CRISPR-Cas9-mediated genome editing was used. The single-guide RNAs (sgRNAs) were designed by using the CRISPR Design tool (Zhang Lab, MIT). For all the genes two separate sgRNAs were designed, and, based on the in vitro screening assay, the better performing sgRNA was chosen for each gene for the following organoid experiments. The sequences of the final sgRNAs are shown in the Table 3.

**Table 3:** Sequences targeted by designed sgRNAs.

Gene	Exon (F—forward, R—reverse)	Targeted sequence
*Trp53*	Exon 3, F	AAGTCACAGCACATGACGG
*Brca1*	Exon 6, F	GCGTCGATCATCCAGAGCGT
*Nf1*	Exon 8, F	CCAGGACATCTCCAAGGATG
*Pten*	Exon 6, R	ATATACATAGCGCCTCTGAC

For organoid transfection experiments about 640 μl of BME with *Cas9*-expressing oviductal or OSE organoids were collected from the plate (about 4/12-wells) and dissociated into single cells using TrypLE, washed with AdDF++ (without antibiotics), resuspended in high-density in 500 μl of growth medium with 10 μM ROCK inhibitor (without antibiotics) and transferred to a well on a 24-well culture plate. The sgRNA transfection was performed by using the Stemfect RNA Transfection Kit (Stemgent) according to the manufacturer′s instructions. Briefly, 12.5 μl of transfection buffer was mixed together with 1 μl of transfection reagent and incubated at room temperature for 10 min followed by addition of the sgRNA mixture containing 12.5 μl of transfection buffer and 5 μg of appropriate sgRNAs. The total volume of transfection mixture (ca 25 μl) was incubated at room temperature for 15 min before adding it to the cell suspension and the plate was placed in a humidified incubator at 37 °C for 4–5 h. After incubation, cells were collected and plated according to the standard protocol and covered with full medium containing 10 μM ROCK inhibitor. Around 2–3 days post transfection, the medium was exchanged with the growth medium containing 10 µM Nutlin-3a (Cayman Chemical) to select for p53 mutant organoids. Within two weeks clonal organoid outgrowth could be readily observed, the organoids were picked, expanded and screened for mutations in targeted genes. If the triple mutants were not obtained after the first round of transfection, an additional transfection was performed.

For genotyping, genomic DNA was isolated using DirectPCR lysis reagent (Viagen). Primers for the PCR amplification using GoTaq Flexi DNA polymerase (Promega) were as follows: Trp53_for, 5′-CAGGAAGCCAAAGGGTGAAGA-3′, Trp53_rev, 5′-CCCATCTACAGTCCCCCTTG-3′; Brca1_for, 5′-TGGAGTGCAAGTGAAAGCCT-3′, Brca1_rev, 5′-ACCGACAATTAAGATGGAGTGCT-3′; Nf1_for, 5′- CCCGGGAAACTATCAGCCTT-3′, Nf1_rev, 5′-CTGTTTGACCTAGCATGGACA-3′; Pten_for, 5′- TGCAGTACAGAGACCATTGACT-3′, Pten_rev, 5′-CGACACACAGACAGCTAAGAA-3′. Products were cloned into pGEM-T Easy vector system I (Promega) and subsequently Sanger sequenced by Macrogen Europe (Amsterdam, The Netherlands) using universal T7 sequencing primer.

### Western blot

Organoids were treated with 10 µM Nutlin-3a to activate p53 pathway 24 h prior to the harvesting. Samples were lysed using RIPA buffer (50 mM Tris-HCl pH 8.0, 150 mM NaCl, 0.1% SDS, 0.5% Na-Deoxycholate, 1% NP-40) containing Complete protease inhibitors (Roche). Protein content was quantified using standard Bradford assay (BioRad) and equal amounts of protein (a′ 20 µg) were run on gradient polyacrylamide gel (4–15%; BioRad) and transferred to PVDF membranes (Millipore). Membranes were blocked and probed with antibodies directed against p53 (sc-6243, 1:250, Santa Cruz Biotechnology) and GAPDH (LN2100751, 1:1000, Labned). Uncropped versions of the western blots are provided in the Supplementary Fig. 6. The results were confirmed twice.

### RNA isolation, cDNA preparation, and qRT-PCR

For qPCR analysis, RNA was isolated from organoids and tissues using the RNeasy Mini Kit (Qiagen, Cat. 74104) following the manufacturer′s instructions including DNaseI treatment. For qPCR, RNA was reverse transcribed from 500 ng of total RNA using GoScript and random primer (both Promega). qPCR was performed with three biological replicates in duplicates using the indicated primers, SYBR Green Mixture (BioRad) and BioRad CFX Manager Version 3.1. Gene expression was quantified using the delta-delta-Ct method and normalized against β-Actin housekeeping gene. qPCR primers used in this study were as follows: β-Actin_for, 5′-GTCGAGTCGCGTCCACC-3′, β-Actin_rev, 5′-GTCATCCATGGCGAACTGGT-3′; Pax8_for, 5′-GATGCCTCACAACTCGATCA-3′, Pax8_rev, 5′-AAGGATCTTGCTTACACAGC-3′; Foxj1_for, 5′-ACCAAGATCACTCTGTCGG-3′, Foxj1_rev, 5′-GATGGAATTCTGCCAGGTG-3′; Dnah5_for, 5′-ATGGACTGACTTCTCGCCTC-3′, Dnah5_rev, 5′-GTCGTTGCGTCAGAACTCG-3′; Trp73_for, 5′-GGGAGCAACAGGCTCTGAAT-3′; Trp73_rev, 5′-GCTCTGCTTGAATGCACGTT-3′.

### Library preparation and RNA-seq analysis

For RNA-seq analysis, RNA was isolated from the organoids and tissues using the RNeasy Mini Kit (Qiagen) following the manufacturer’s instructions or standard Tryzol extraction protocol, respectively. In both cases DNaseI treatment was included. In vitro transcription was performed using 1–5 ng cDNA as template and RNA was reverse transcribed into a sequencing library. After preparation, the quality and quantity of the libraries were checked with Bioanalyzer2100 DNA High Sensitivity chips (Cat. 5067-4626) and Qubit (Qubit^®^ dsDNA HS Assay Kit, Cat. Q32854); all samples had a RIN value of 10. Sequencing was performed on an Illumina NextSeq500 by using 75-bp paired-end sequencing. Paired-end reads from Illumina sequencing were aligned to the mouse genome (GRCm38 assembly) with BWA^55^. The raw data file consists of a total number of reads for each gene (without UMI correction) that were uniquely mapped to the transcriptome (with a mapping quality above 60), and that had the appropriate transcription direction. DESeq2 (v1.18.0) package was used to normalize count data and for differential gene expression analysis in program R (R version 3.5.1, Bioconductor version 3.8 (BiocManager 1.30.4)). Gene set enrichment analysis (GSEA) was performed using GSEA software v3.0 beta2.

### In vitro drug screen

Two days prior to the start of the assay, organoids were disrupted into single cells using TrypLE and filtered using a 70-μm nylon cell strainer (BD Falcon). The cells were subsequently counted, and resuspended in 5% BME/growth medium (25,000 cells/mL) prior plating in 40 μl volume (Multi-drop Combi Reagent Dispenser, Thermo Scientific, catalog no. 5840300) in 384-well plates (Corning, catalog no. 4588). The drugs were added 2 days after plating the cells using the Tecan D300e Digital Dispenser (Tecan). Nutlin-3a (Cayman Chemical, catalog no. 10004372), niraparib (Selleckchem, catalog no. S2741), and paclitaxel (Sigma, catalog no. T7402) were dissolved in DMSO. Cisplatin (Sigma, catalog no. C2210000) was dissolved in PBS containing 0.3% Tween-20, which was required to dispense the drug using the HP printer. All wells were normalized for the solvent used. DMSO percentage never exceeded 1% and PBS/Tween-20 percentage never exceeded 2%. Drug exposure was performed in quadruplicates for each concentration shown. Five days (120 h) after the addition of the drugs, ATP levels were measured using the CellTiter-Glo 3-D Reagent (Promega, catalog no. G9681) according to the manufacturer′s instructions, and luminescence was measured using a Spark multimode microplate reader (Tecan). Results were normalized to vehicle (100%) and baseline control (Staurosporine 1 μmol/L; 0%). Data were analyzed using GraphPad Prism software (version 7.04) and lines were fitted using the option “log(inhibitor) vs normalized response–variable slope”. Drug screening results were confirmed in quadruplicates (*n* = 4) over two independent experiments.

### In vivo transplantation assays

Before transplantations, wild-type or mutant oviductal and OSE organoids were harvested and broken into smaller injectable fragments via mechanical shearing with a fire-polished glass pipette. Cells were then resuspended in 10% BME in PBS and ~100,000 cells were injected per location. Both subcutaneous and orthotopic injections were performed. For each mutation combination, two separate clones were transplanted. At least two mice were used for orthotopic and one mouse for subcutaneous transplantations per clone. Ear clipping was used for animal recognition. The mice were sacrificed up to 4 months (ca 120 days) after injections. Tumor volumes were measured and estimated by formula: Tumor volume = (length × width^2^)/2, where length represents the largest tumor diameter and width the perpendicular tumor diameter. Tumor volume was measured on all the OSE-derived tumors and randomly selected oviductal-derived tumors. All the tumors were subjected to immunohistochemical analysis. KI67- and cleaved Caspase-3-positive cells were quantified using ImageJ software. From *n* = 4 tumors also organoids were derived.

### Establishment of tumor-derived organoids

A small piece of a tumor tissue was dissected, mechanically dissociated and cells were extracted by collagenase treatment as described in the Methods above (same protocol as under Derivation of oviductal organoids). Cultures were grown under Nutlin-3a selection to specifically promote the outgrowth of the tumor cells and inhibit the growth of host cells.

### Reporting summary

Further information on research design is available in the Nature Research Reporting Summary linked to this article.

## Supplementary information

Supplementary Information

Description of Additional Supplementary Files

Supplementary Video 1

Supplementary Data 1

Supplementary Data 2

Supplementary Data 3

Supplementary Data 4

Reporting Summary

## Data Availability

The RNA-sequencing data have been deposited in the GEO database under the accession code GSE147882. The gene signature lists for different molecular subtypes of HG-SOC referenced during the study are available under Konecny et al. supplementary data at 10.1093/jnci/dju249. The source data underlying Figs. 1–3, 5, and Supplementary Figs. 1–4 and 6 are provided as a Source data file. All the other data supporting the findings of this study are available within the article and its supplementary information files and from the corresponding author upon reasonable request. A reporting summary for this article is available as a Supplementary Information file.

## Source Data

Source Data
