# Supplementary material for: Assessing the origin of high-grade serous ovarian cancer using CRISPR-modification of mouse organoids
Source: Nat Commun. 2020 May 27;11:2660. doi: 10.1038/s41467-020-16432-0 (PMC7253462; doi:10.1038/s41467-020-16432-0)
Supplement: Supplementary file 1 — Supplementary Information [file 41467_2020_16432_MOESM1_ESM.pdf]

Supplementary Information

Assessing the origin of high-grade serous ovarian cancer using CRISPR-modification of mouse organoids

Lõhmussaar et al.

Supplementary Figures

a

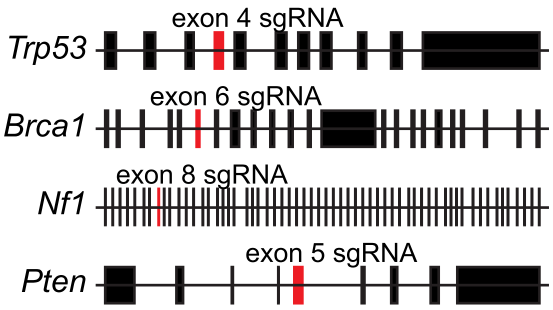

	Trp53	*Brca1*	*Nf1*	*Pten*
# of clones screened	10	123	14	24
# of clones not targeted	0*	104	3	9
# of clones targeted	10	19	11	15
One allele targeted (FS)	0	13	2	4
One allele targeted (IF)	0	3	0	1
Both alleles targeted (FS)	5	0	8	8
Both alleles targeted (IF)	5	3	1	2
Total percent of targeted	100%	15%	79%	63%

*Nutlin-3a selection

b

Oviduct clones	*Trp53*	*Brca1*	*Nf1*	*Pten*
Ovi-T1	(+1/+1)	-	-	-
Ovi-T2	(-19/-19)	-	-	-
Ovi-TB1	(-1/+1)	(wt/-2)	-	-
Ovi-TB2	(-19/-19)	(wt/+1)	-	-
Ovi-TBN1	(-50/-50)	(wt/-26)	(-4/-4)	-
Ovi-TBN2	(-19/-19)	(wt/+1)	(-1/-4)	-
Ovi-TBP1	(-50/-50)	(wt/-26)	-	(-2/-2)
Ovi-TBP2	(-19/-19)	(wt/+1)	-	(+1/+1)

OSE clones	*Trp53*	*Brca1*	*Nf1*	*Pten*
OSE-T1	(-1/-10)	-	-	-
OSE-T2	(-1/-4)	-	-	-
OSE-TB1	(-1/-10)	(+1/-12)	-	-
OSE-TB2	(-1/-10)	(-2/-12)	-	-
OSE-TBN1	(-1/-2)	(-2/-3)	(-1/-1)	-
OSE-TBN2	(+1/-10)	(-2/-12)	(-1/-1)	-
OSE-TBP1	(+1/-10)	(-2/-12)	-	(-5/-8)
OSE-TBP2	(+1/-10)	(-2/-12)	-	(+1/-5)

c

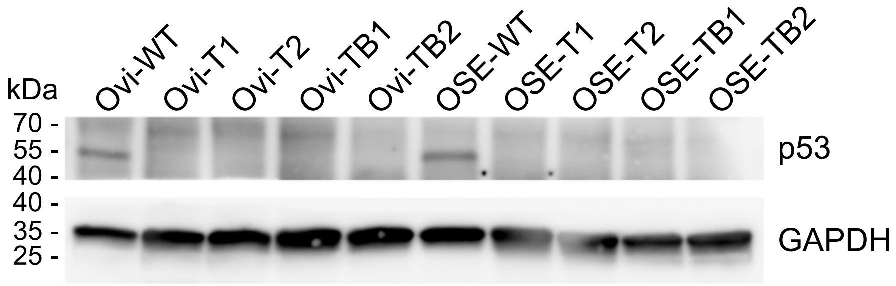

d

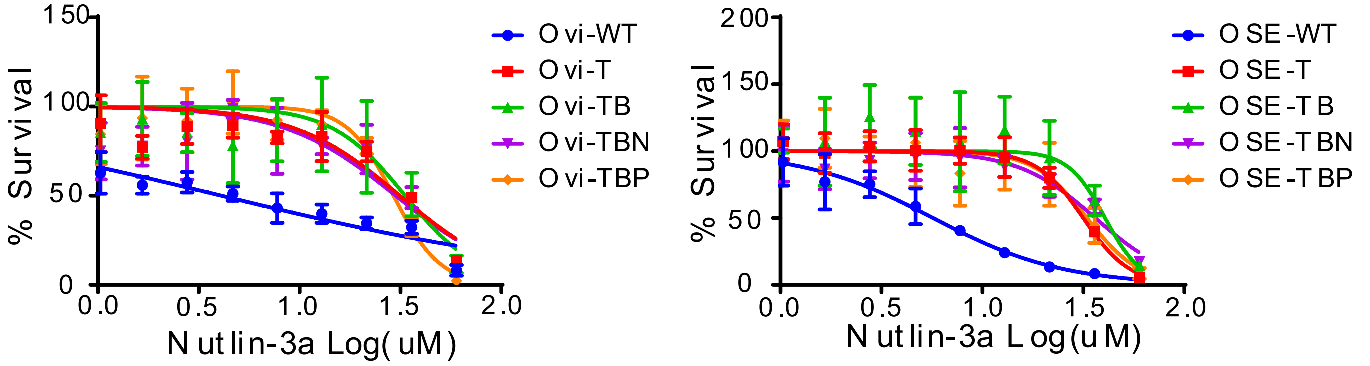

Supplementary Figure 1. Derivation of mutant clones via CRISPR-Cas9. (a) The sgRNA targeting exons and their targeting efficiency in the indicated genes. Asterisk - followed by Nutlin-3a selection 100% of the clones were targeted in *Trp53* gene. **(b)** Summary tables of all the established clones and their exact mutations from oviductal (top) and OSE (bottom) origin. **(c)** Western blot analysis of p53 expression in wild-type, T- and TB-mutant organoids from both lineages. GAPDH is shown as a loading control. Representative from n=2 independent experiments. Uncropped images of the blots are provided in the Supplementary Figure 6. **(d)** Nutlin-3a sensitivity assay of mutants and respective wild-types from oviductal (left graph) and OSE (right graph) lineages. Dots and error bars represent the mean and ±SEM of technical quadruplicates (n=4), respectively, over two independent experiments. **(c-d)** Ovi - oviduct; WT - wild-type; T – *Trp53* mutant; TB – *Trp53*, *Brca1* mutant; TBN – *Trp53*, *Brca1*, *Nf1* mutant; TBP – *Trp53*, *Brca1*, *Pten* mutant.

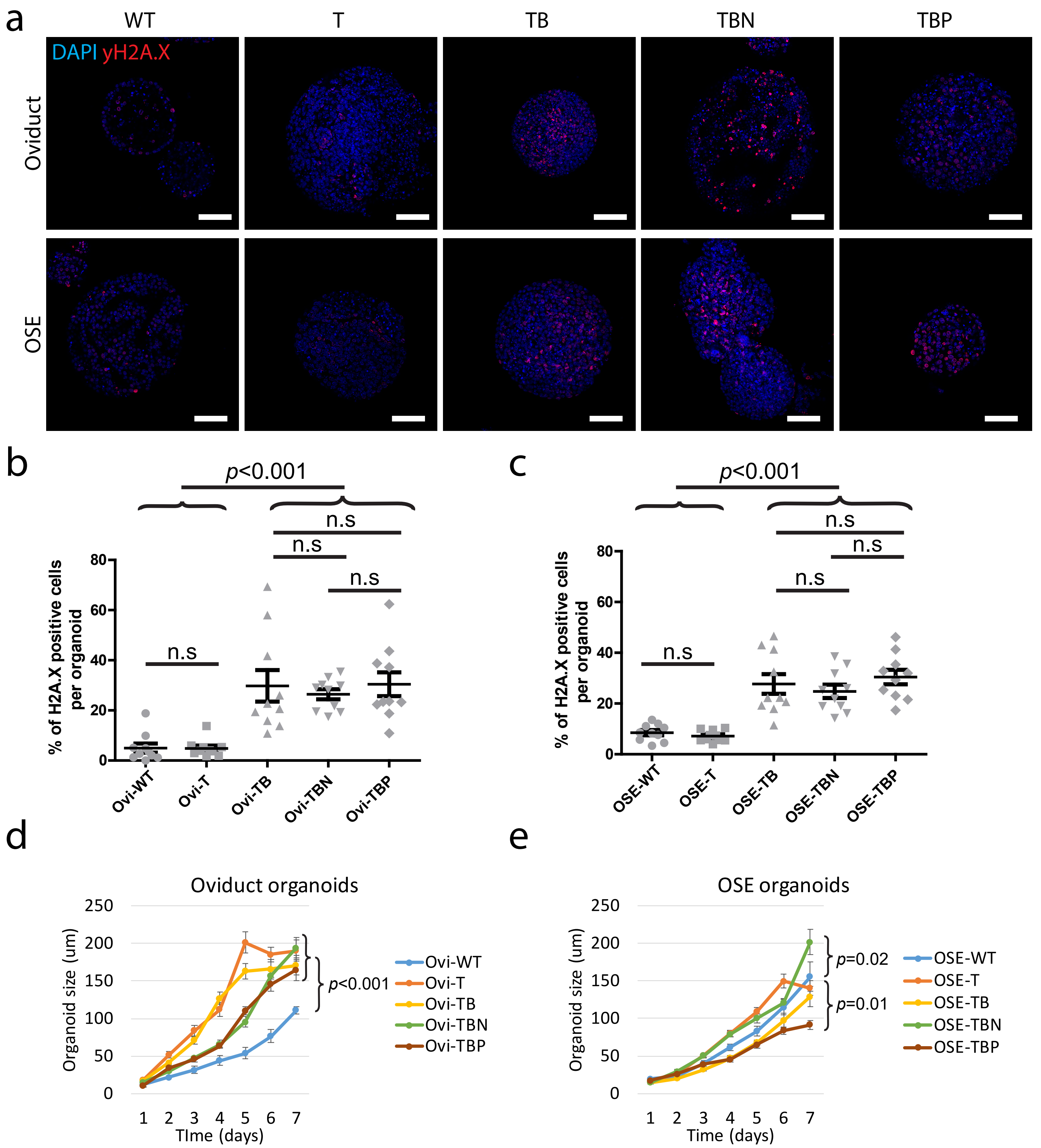

f

Clone	Live (Annexin V-/PI-)	Early apoptotic (Annexin V+/PI-)	Late apoptotic (Annexin V+/PI+)
Ovi-WT	53.4%	38.2%	4.2%
Ovi-T	70.2%	24.4%	2.3%
Ovi-TB	66.7%	27.3%	2.8%
Ovi-TBN	79.8%	16.7%	1.3%
Ovi-TBP	80.1%	16.2%	1.2%
OSE-WT	53.7%	27.6%	8.9%
OSE-T	87.1%	7.3%	1.0%
OSE-TB	76.7%	17.6%	1.7%
OSE-TBN	71.8%	21.7%	2.9%
OSE-TBP	43.6%	48.1%	6.3%

Supplementary Figure 2. Additional characterization of mutant clones. (a) Representative images of DNA damage induction in clones from both lineages after overnight treatment with Mitomycin C, measured by γH2A.X immunofluorescence (n=2 independent experiments). Scale bar, 100 μm. **(b-c)** Quantification of **(a)**: Percentage of nuclei positive for γH2A.X in Mitomycin C-treated organoids. Error bars represent ±SEM (n=10 organoids/line). Statistical significance was calculated by two-tailed Student's t-test, *p*-values were not adjusted for multiple comparisons, n.s - not significant. **(d-e)** Organoid growth assay measured by daily increase in organoid sizes in oviductal **(d)** and OSE **(e)** lineages for a week (n=2 independent experiments). Statistical significance was calculated by two-tailed Student's t-test, *p*-values were not adjusted for multiple comparisons. Error bars represent ±SEM (n=12 organoids/line). **(f)** Percentages of cells stained for Annexin V and PI and analysed by flow cytometry to evaluate apoptosis in all clones (n=2 independent experiments). **(a-f)** Ovi - oviduct; WT – wild-type, T – *Trp53* mutant; TB – *Trp53*, *Brca1* mutant; TBN – *Trp53*, *Brca1*, *Nf1* mutant; TBP – *Trp53*, *Brca1*, *Pten* mutant.

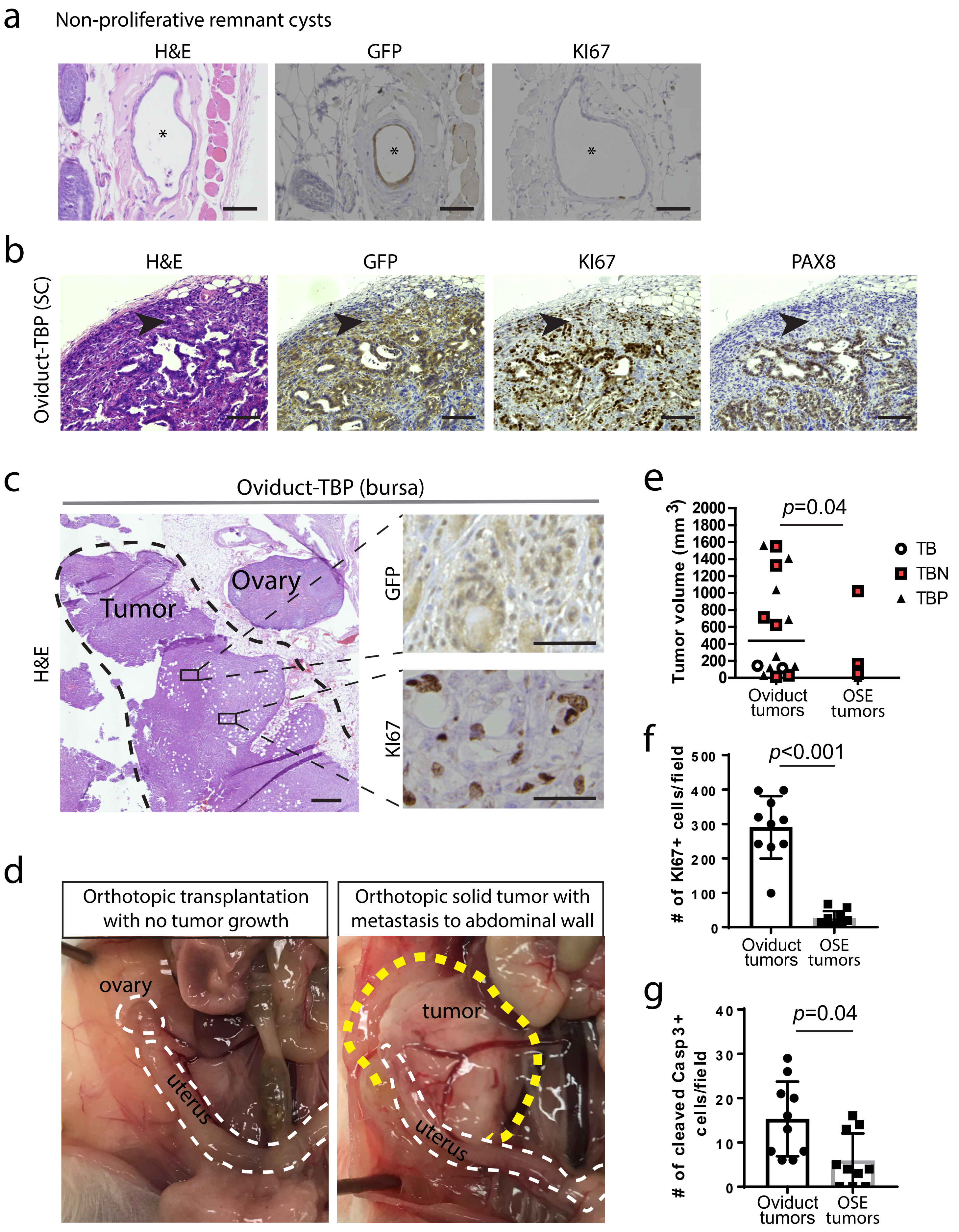
a
Non-proliferative remnant cysts
H&E
GFP
KI67
b
Oviduct-TBP (SC)
H&E
GFP
KI67
PAX8
c
Oviduct-TBP (bursa)
H&E
Tumor
Ovary
GFP
KI67
d
Orthotopic transplantation with no tumor growth
ovary
uterus
Orthotopic solid tumor with metastasis to abdominal wall
tumor
uterus
e
Tumor volume (mm 3)
p=0.04
TB
TBN
TBP
Oviduct tumors
OSE tumors
f
# of KI67+ cells/field
p<0.001
Oviduct tumors
OSE tumors
g
# of cleaved Casp3+ cells/field
p=0.04
Oviduct tumors
OSE tumors

Supplementary Figure 3. Additional characterization of organoid-derived tumors. (a) Representative histological stainings of non-proliferative remnant cysts (asterisks) observed in subcutaneous transplantation with the wild-type organoids (n=4 injections). Scale bar, 50 μm. **(b)** Histological example of an oviduct-TBP clone-derived subcutaneous tumor showing epithelio-mesenchymal phenotype. H&E, GFP, KI67 and PAX8 stainings are shown (n=4 mice observed). Arrow heads point to the GFP-positive mesenchymal-like cells that have lost the expression of PAX8. Scale bar 100 μm. **(c)** Representative histological stainings of orthotopic solid tumor derived from oviductal TBP clone (n=8 tumors). H&E, GFP and KI67 stainings are shown. H&E staining scale bar, 500 μm; GFP/KI67 image scale bar, 50 μm. **(d)** Representative images of orthotopic transplantations with oviductal clones which yielded no tumor (left, n=23 injections) or solid tumor (right, n=15 injections) growth with abdominal wall metastases. Uterus horn and ovary – white dashed line, tumor – yellow dashed line. **(e)** The distribution and mean of the tumor volumes derived from oviduct (n=16) and OSE (n=7). Statistical significance was calculated by one-sided unpaired Student's t-test. **(f)** Number of KI67-positive cells per 20x magnification image fields in oviduct- and OSE-derived tumors (5 fields per tumor, 2 tumors/origin, n=10). Error bars represent ±SEM. Statistical significance was calculated by two-tailed Student's t-test. **(g)** Number of cleaved Caspase-3 positive cells per 20x magnification image fields in oviduct- and OSE-derived tumors (5 fields per tumor, 2 tumors/origin, n=10). Error bars represent ±SEM. Statistical significance was calculated by two-tailed Student's t-test.

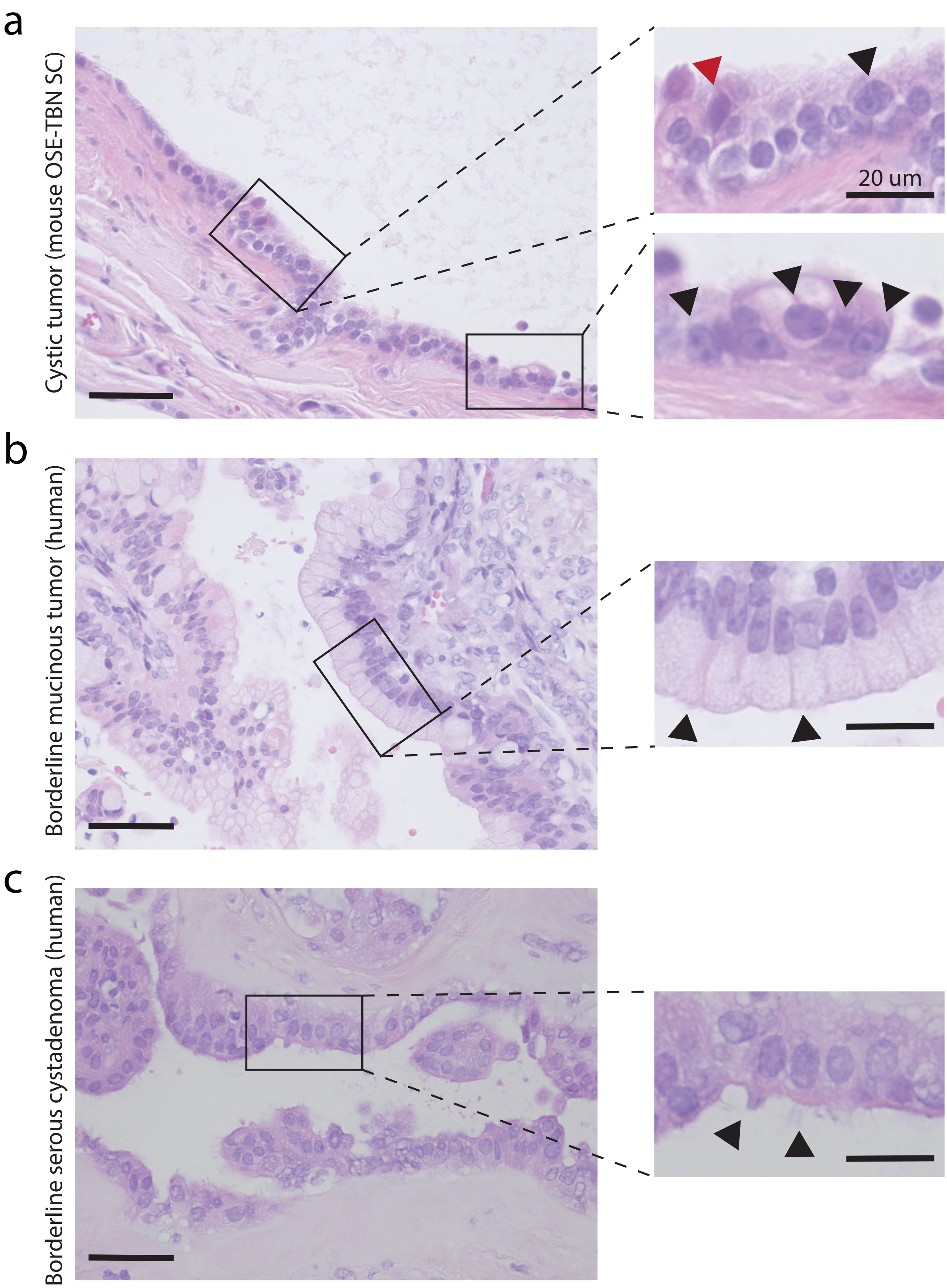

Supplementary Figure 4. Comparative histological properties of a murine organoid-derived cystic tumor and two distinct human benign ovarian tumors. (a) Representative image of a murine organoid-derived cystic tumor (n=9 mice observed). As an example, OSE-TBN (*Trp53, Brca1, Nf1* mutant) clone-derived subcutaneous (SC) tumor is shown. Upper inset: multiple nucleoli (black arrowhead) and nuclear atypia (red arrowhead). Bottom inset: abundant mitotic figures (black arrowheads). **(b)** Human borderline mucinous tumor from a patient. Inset: mucinous glands (arrowheads). **(c)** Human borderline serous cystadenoma from a patient. Inset: cilium (arrowheads). **(a-c)** Large image scale bar, 50 μm; inset scale bar, 20 μm.

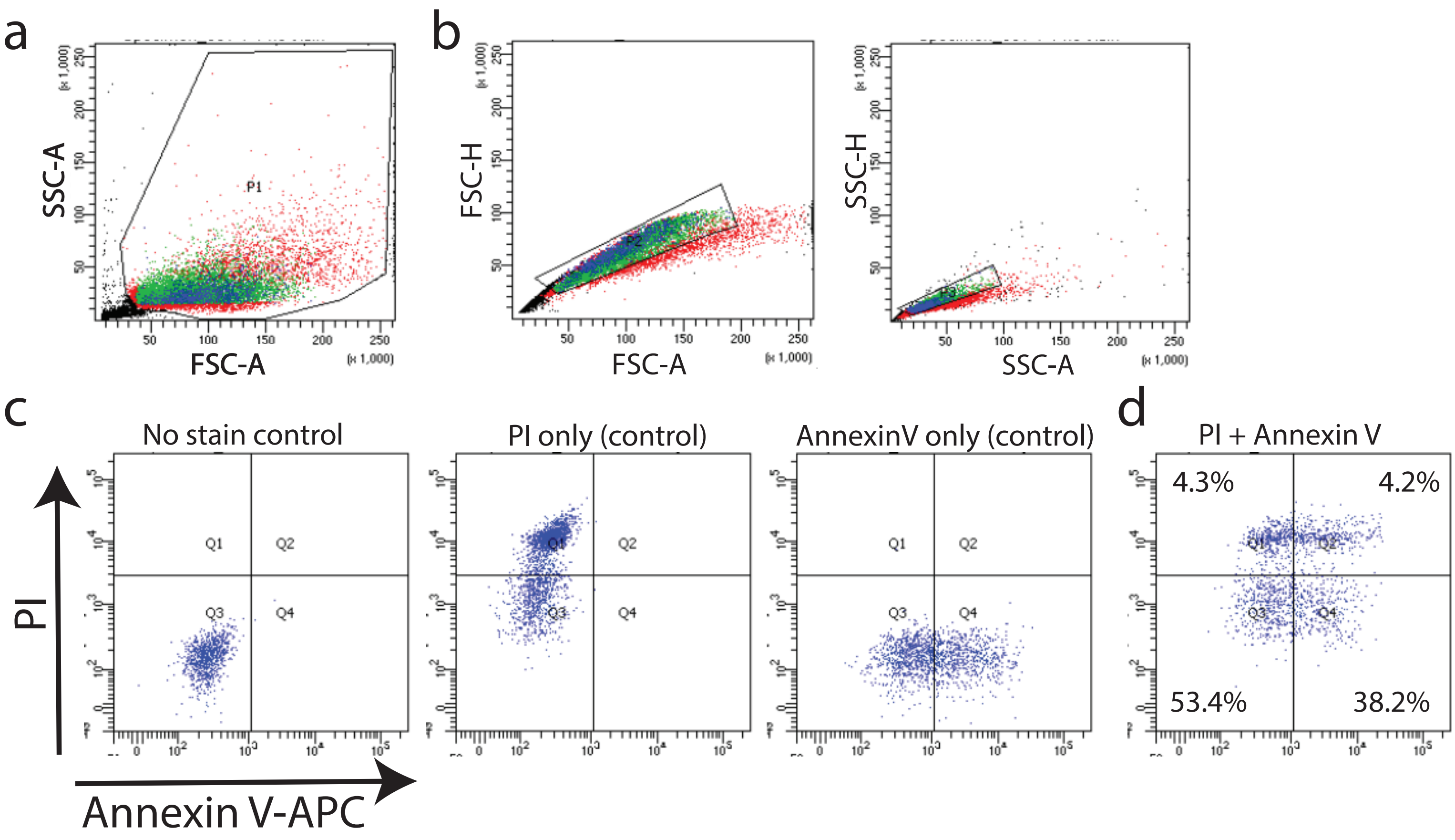

Supplementary Figure 5. FACS gating strategy. (a) In this sample gating, the cells were first gated for forward- and side-scatter area (FSC-A vs SSC-A) to select the cell population of interest and exclude the debris. **(b)** Next, a sequential gating was performed to obtain single cells. The cells were first gated for forward-scatter area and height (FSC-A vs FSC-H) followed by gating for side-scatter area and height (SSC-A vs SSC-H), which allows for higher sensitivity in doublet exclusion. **(c)** No stain, "PI only" and "Annexin V only" samples were used to set up the gates for the assay. **(d)** Subsequently, PI and Annexin V-APC double-stained clones were analysed for apoptotic events. Q1: PI-positive and Annexin V-negative necrotic cell fraction, Q2: PI and Annexin V double-positive late apoptotic cell fraction; Q3: PI- and Annexin V-negative live cell fraction; Q4: PI-negative and Annexin V-positive early apoptotic cells. The main results are shown in the Supplementary Figure 2f.

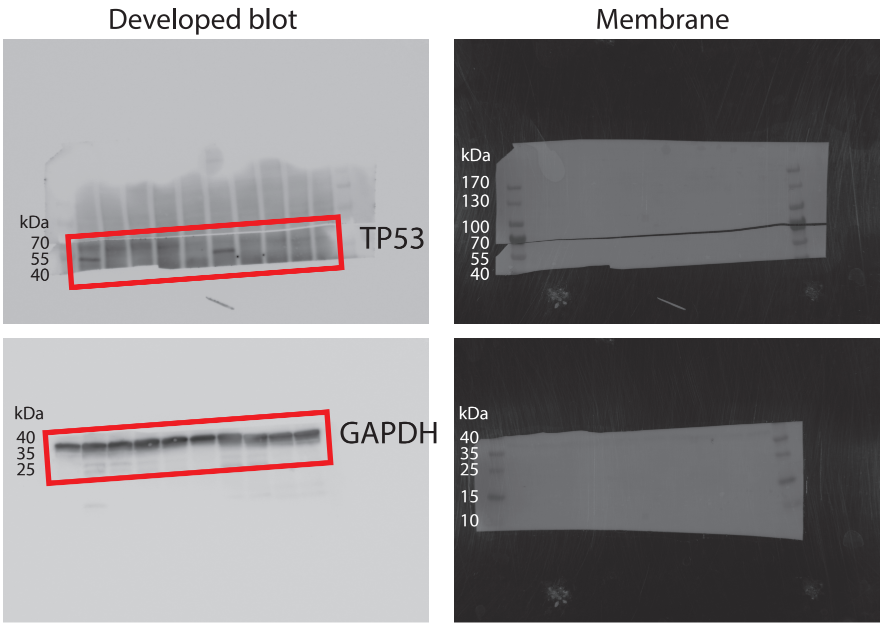

Supplementary Figure 6. Uncropped images of Western blots. The panels circled with red rectangles are displayed in the Supplementary Figure 1c.
