# Supplementary material for: Assessing the origin of high-grade serous ovarian cancer using CRISPR-modification of mouse organoids
Source: Nat Commun. 2020 May 27;11:2660. doi: 10.1038/s41467-020-16432-0 (PMC7253462; doi:10.1038/s41467-020-16432-0)
Supplement: Supplementary file 2 — Description of Additional Supplementary Files [file 41467_2020_16432_MOESM2_ESM.docx]

File name: Supplementary Video 1

Description: High speed time-lapse imaging of normal oviductal organoid line with beating cilia. Beating cilia border the luminal side of the organoid wall.

File name: Supplementary Data 1

Description: Medium recipes of mouse oviductal and OSE organoids (for total of 50 ml).

File name: Supplementary Data 2

Description: Statistical analysis of targeting odds on *Brca1* and *Pten* locuses.

File name: Supplementary Data 3

Description: Transplantation outcomes with oviduct-derived clones.

File name: Supplementary Data 4

Description: Transplantation outcomes with OSE-derived clones.
