# Supplementary material for: Assessing the origin of high-grade serous ovarian cancer using CRISPR-modification of mouse organoids
Source: Nat Commun. 2020 May 27;11:2660. doi: 10.1038/s41467-020-16432-0 (PMC7253462; doi:10.1038/s41467-020-16432-0)
Supplement: Supplementary file 8 — Reporting Summary [file 41467_2020_16432_MOESM8_ESM.pdf]

nature research

Corresponding author(s): Hans Clevers

Last updated by author(s): Apr 1, 2020

Reporting Summary

Nature Research wishes to improve the reproducibility of the work that we publish. This form provides structure for consistency and transparency in reporting. For further information on Nature Research policies, see Authors & Referees and the Editorial Policy Checklist .

Statistics

For all statistical analyses, confirm that the following items are present in the figure legend, table legend, main text, or Methods section.

n/a	Confirmed	
☐	☒	The exact sample size (*n*) for each experimental group/condition, given as a discrete number and unit of measurement
☐	☒	A statement on whether measurements were taken from distinct samples or whether the same sample was measured repeatedly
☐	☒	The statistical test(s) used AND whether they are one- or two-sided<br>*Only common tests should be described solely by name; describe more complex techniques in the Methods section.*
☒	☐	A description of all covariates tested
☒	☐	A description of any assumptions or corrections, such as tests of normality and adjustment for multiple comparisons
☐	☒	A full description of the statistical parameters including central tendency (e.g. means) or other basic estimates (e.g. regression coefficient) AND variation (e.g. standard deviation) or associated estimates of uncertainty (e.g. confidence intervals)
☐	☒	For null hypothesis testing, the test statistic (e.g. *F*, *t*, *r*) with confidence intervals, effect sizes, degrees of freedom and *P* value noted<br>*Give P values as exact values whenever suitable.*
☒	☐	For Bayesian analysis, information on the choice of priors and Markov chain Monte Carlo settings
☒	☐	For hierarchical and complex designs, identification of the appropriate level for tests and full reporting of outcomes
☒	☐	Estimates of effect sizes (e.g. Cohen's *d*, Pearson's *r*), indicating how they were calculated

Our web collection on statistics for biologists contains articles on many of the points above.

Software and code

Policy information about availability of computer code

Data collection

Leica LAS X Version 1.1, Bio-Rad CFX Manager Version 3.1

Data analysis

Libraries were sequenced on an Illumina NextSeq500 by using 75-bp paired-end sequencing. Paired-end reads from Illumina sequencing were aligned to the mouse genome (GRCm38 assembly) with BWA (Li and Durbin 2009). The raw datafile consists of a total number of reads for each gene (without UMI correction) that were uniquely mapped to the transcriptome (with a mapping quality above 60), and that had the appropriate transcription direction. DESeq2 (v1.18.0) package was used to normalize count data and for differential gene expression analysis in program R (R version 3.5.1, Bioconductor version 3.8 (BiocManager 1.30.4)). Gene set enrichment analysis (GSEA) was performed using GSEA software v3.0 beta2.

qPCR data was analyzed in Microsoft Excel 2019 using delta-delta Ct method.

Organoid sizes were measured using ImageJ software (version 1.51j8).

Immunohistochemistry samples were imaged on DM4000 light microschope using LAS X software (Version 1.1) and processed using ImageJ (Version 1.51p). Immunofluorescent stainings for yH2A.X were imaged on a Leica SP8 confocal microscope, and positive cells quantified by manual counting.

Drug screening kill curves were produced using GraphPad Prism software (version 7.04) and lines were fitted using the option 'log (inhibitor) vs normalized response -variable slope'.

For manuscripts utilizing custom algorithms or software that are central to the research but not yet described in published literature, software must be made available to editors/reviewers. We strongly encourage code deposition in a community repository (e.g. GitHub). See the Nature Research guidelines for submitting code & software for further information.

Data

Policy information about availability of data

All manuscripts must include a data availability statement. This statement should provide the following information, where applicable:

- Accession codes, unique identifiers, or web links for publicly available datasets
- A list of figures that have associated raw data
- A description of any restrictions on data availability

The RNA-sequencing data have been deposited in the GEO database under the accession code GSE147882. The gene signature lists for different molecular subtypes of HG-SOC referenced during the study are available under Konecny et al. (2014) supplementary data at doi: 10.1093/jnci/dju249. The source data underlying Figures 1-3, 5 and Supplementary Figures 1-4, 6 are provided as a Source Data file. All the other data supporting the findings of this study are available within the article and its supplementary information files and from the corresponding author upon reasonable request. A reporting summary for this article is available as a Supplementary Information file.

Field-specific reporting

Please select the one below that is the best fit for your research. If you are not sure, read the appropriate sections before making your selection.

[x] Life sciences [ ] Behavioural & social sciences [ ] Ecological, evolutionary & environmental sciences

For a reference copy of the document with all sections, see nature.com/documents/nr-reporting-summary-flat.pdf

Life sciences study design

All studies must disclose on these points even when the disclosure is negative.

Sample size

Ovaries and oviducts from at least 6 mice were used to establish a single oviductal and OSE organoid line. The number of mice (n=6) for one experiment was chosen due to small amount of epithelial cells that could be derived from the oviducts and ovaries of a single mice. At least 4 independent organoid lines were established per origin.

qPCR experiment was performed on n=3 independent biological replicates with two technical replicates per each sample. The experiment was repeated three times. This sample size was chosen to confirm that our results are line-independent and reproducible.

RNA-seq analysis was performed on 3 independent tissue samples or organoid lines, except for OSE organoid lines where 1 of the 3 lines was exluded from analysis due to evident contamination. This sample size was chosen to confirm that our results are line-independent and reproducible. Additionally, RNA-sequencing was performed on 6 independent tumor tissues derived from subcutaneously grown tumors. This sample size was chosen to reliably characterize the tumors with statistically significant power.

Two independent organoid clones were analyzed per each mutation or their combination per tissue of origin to guarantee representative results. Each clone was transplanted into at least 3 immunodeficient mice, the injections were done to both sides of each mice (left/right flank or left/right ovarian bursa in subcutaneous and orthotopic transplantations, respectively). At least 2 mice were used for orthotopic transplantations per clone (2 injected ovaries/mouse) and at least 1 mouse was used for subcutaneous transplantations per clone (2 flanks/mouse). This strategy was favored due to the previous knowledge that orthotopic transplantations will better mimic the original micro-environment of the tumor and orthotopic tumors will therefore recapitulate the disease better. However, as the subcutaneous transplantations are easier to perform and have a higher success rate compared to orthotopic injections, one mouse per clone was assigned for receiving a subcutaneous injection to ensure the success of the overall experiment. Altogether, each clone was injected to in total of 6 locations (4 orthotopic + 2 subcutaneous) and two independent clones were transplanted per mutation, therefore, the tumorigenic effect of each mutation type was evaluated in in total of 12 locations/6 mice. This set-up guaranteed the success of the experiment and provided statistically significant data on the effect of the mutation type and tissue of origin to the tumor development.

Organoid sizes were calculated based on the measurements taken from 12 organoids per line.

In drug screening assays the drug exposure was performed in quadruplicates (n=4) for each concentration over 2 independent experiments.

Data exclusions

One OSE organoid line was excluded from analysis since there was evident contamination.

Replication

Organoid establishment: at least 4 independent organoid lines were successfully established per origin.
qPCR data: The organoid differenatiation assay was confirmed over 3 independent experiments.
Growth assay: the diameter of 12 organoids/clone were measured. Assay was replicated twice.
Karyotyping: at least 15 spreads were counted per line and in many cases more.
yH2A.X quantification: at least 10 organoids were quantified per line for positive staining. Two independent experiments were performed.
KI67- and cleaved Caspase-3 quantification: marker-positive cells per 20x magnification field were quantified (5 fields/tumor, 2 tumors/origin)
Drug screens: drug screening results were confirmed in quadruplicates (n=4) over two independent experiments.
FACS assay: two independent experiment were performed to confirm the apoptotic characteristics of the lines.

Randomization

For organoid derivation experiments, a random cohort of wild-type C57BL/6 or CRISPR/CAS9 knock-in (B6J.129(B6N)-Gt(ROSA)26Sortm1(CAG-cas9*,-EGFP)Fezh/J) female mice (between 8-12 weeks) were used to obtain OSE and oviductal tissue.
For differentiation assay, the organoids were splitted and evenly divided over the wells for the DAPT treatment assay.

For RNA-seq analysis, bulk RNA was extracted from 2-3 drops of BME with organoids (per line) in order to provide sufficient material for library preparation.
In the drug-screening assay, the organoids were trypsinized into single-cell suspension and randomly distributed throughout the screening plate using automated dispenser.
For the transplantation experiment, all the female mice were randomly allocated into 10 groups - 1) Control oviduct wild-type organoids, 2) Oviduct single mutants, 3) Oviduct double mutants, 4) Oviduct triple mutants (TBN), 5) Oviduct triple mutants (TBP), 6) Control OSE wild-type organoids, 7) OSE single mutants, 8) OSE double mutants, 9) OSE triple mutants (TBN), 10) OSE triple mutants (TBP).
For tumor RNA-seq analysis, 6 random oviductal TBP-derived tumors were included.

Blinding

Blinded evaluation of tumors was performed by expert pathologist.
RNA-seq initial analysis was carried out as a blinded experiment to visualize the overall sample characteristics without allocating data to any distinct groups. During the further analysis investigators were not blinded as the interest was to find out clear differences between distinct groups.
No blinding was performed for other experiments as standard protocols were equally applied on all samples.

Reporting for specific materials, systems and methods

We require information from authors about some types of materials, experimental systems and methods used in many studies. Here, indicate whether each material, system or method listed is relevant to your study. If you are not sure if a list item applies to your research, read the appropriate section before selecting a response.

Materials & experimental systems

n/a	Involved in the study
	☒ Antibodies
	☒ Eukaryotic cell lines
☒	☐ Palaeontology
	☒ Animals and other organisms
	☒ Human research participants
☒	☐ Clinical data

Methods

n/a	Involved in the study
☒	☐ ChIP-seq
	☒ Flow cytometry
☒	☐ MRI-based neuroimaging

Antibodies

Antibodies used

Antibodies used:
Mouse-anti-Cytokeratin-8 antibody (clone number Ks8.7) from Santa Cruz (1:50). Catalogue number: sc-101459, monoclonal. Lot #G0114
Rabbit-anti-Pax8 antibody from Proteintech (1:2000). Catalogie number: 10336-1-AP, polyclonal. Lot #00019427
Mouse-anti-Human Ki67 antibody (clone number MM1) from Monosan (1:2000). Catalogue number: MONX10283, monoclonal. No lot available.
Mouse-anti-acetylated a Tubulin antibody (clone number: 6-11B-1) from Santa Cruz (1:2000). Catalogue number: sc-23950, monoclonal. Lot #K1317
Rabbit-anti-GFP from Life Technologies (1:1000). Catalogue number: A11122, polyclonal. Lot #1925070
Rabbit-anti-cleaved-Caspase-3 (D175) from Cell Signaling Technology (1:500). Catalogue number: #9661, polyclonal. Lot #43
Mouse-anti-phospho-histone H2A.X (Ser139) antibody (clone JBW301) from Millipore (1:500). Catalogue number: 05-636. Lot: #3292608
Goat-anti-mouse AF-647 from Thermo Fisher (1:250). Catalogue number: A-21235, polyclonal. Lot #1608485
Rabbit anti-P53 (FL-393) from Santa Cruz (1:250). Catalogue number: sc6243, polyclonal. Lot #H0415
Rabbit anti-GAPDH UNLB (unlabelled, purified) from Labned (1:1000). Catalogue number: LN2100751. Lot #18/05-G4-C5

Validation

All antibodies were used against mouse tissue, have species reactivity on mouse and are applicable to the corresponding assays (IHC/IF/WB) used as validated by the supplier. Owing to the long-term experience with organoid technology, working protocols for organoid stainings have been previously established for majority of the antibodies described in this manuscript in our lab and have been cited below.

Mouse-anti-Cytokeratin-8 antibody (IHC, 1:50, overnight RT, Citrate buffer, pH 6.0/ Santa Cruz, sc-101459)
https://www.scbt.com/p/cytokeratin-8-antibody-ks8-7
Lab ref (organoids): Nat Med. 2019 May;25(5):838-849. doi: 10.1038/s41591-019-0422-6

Rabbit-anti-Pax8 antibody (IHC, 1:000, overnight RT, Citrate buffer, pH 6.0/ Proteintech, 10336-1-AP)
https://www.ptglab.com/products/PAX8-Antibody-10336-1-AP.htm
Lab ref (organoids): Nat Biotechnol. 2019 Mar;37(3):303-313. doi: 10.1038/s41587-019-0048-8

Mouse-anti-Human Ki67 antibody (IHC, 1:2000, overnight RT, Citrate buffer, pH 6.0 (autoclave!)/ Monosan, MONX10283)
https://www.labome.com/product/Cell-Sciences/MONX10283.html
Lab ref (organoids): Proc Natl Acad Sci U S A. 2019 Mar 5;116(10):4567-4574. doi: 10.1073/pnas.1803595116

Mouse-anti-acetylated a Tubulin antibody (IHC, 1:2000, overnight RT, Citrate buffer, pH 6.0,/Santa Cruz, sc-23950)
https://www.scbt.com/p/acetylated-alpha-tubulin-antibody-6-11b-1
Lab ref (organoids): EMBO J. 2019 Feb 15; 38(4): e100300. doi: 10.15252/embj.2018100300

Rabbit-anti-GFP (IHC, 1:1000, overnight RT, Citrate buffer, pH 6.0/ Life Technologies, A11122)
https://www.thermofisher.com/antibody/product/GFP-Antibody-Polyclonal/A-11122
Lab ref (organoids): EMBO J. 2019 Feb 15; 38(4): e100300. doi: 10.15252/embj.2018100300

Rabbit-anti-cleaved-Caspase-3 (IHC, 1:500, overnight RT, Citrate buffer, pH 6.0/ Cell Signaling Technology, #9661)
https://www.cellsignal.com/products/primary-antibodies/cleaved-caspase-3-asp175-antibody/9661
Lab ref (organoids): Nat Cell Biol. 2020 Mar;22(3):321-331. doi: 10.1038/s41556-020-0472-5

Mouse-anti-phospho histone H2A.X (whole mount IF, 1:500, overnight 4ºC/ Millipore, 05-636)
https://www.merckmillipore.com/NL/en/product/Anti-phospho-Histone-H2A.X-Ser139-Antibody-clone-JBW301,MM_NF-05-636?ReferrerURL=https%3A%2F%2Fwww.google.com%2F
Lab ref (organoids): Nature. 2020 Feb 27. doi: 10.1038/s41586-020-2080-8

Goat-anti-mouse AF-647 (whole-mount IF, 1:250, 2 hours RT/ Thermo Fisher, A21235)
https://www.thermofisher.com/antibody/product/Goat-anti-Mouse-IgG-H-L-Cross-Adsorbed-Secondary-Antibody-Polyclonal/A-21235
Lab ref: Cell. 2020 Jan 23;180(2):233-247.e21. doi: 10.1016/j.cell.2019.11.038

Rabbit anti-P53 (WB, 1:250, overnight 4ºC/ Santa Cruz, sc6243) This antibody has been discontinued!
https://www.scbt.com/p/p53-antibody-fl-393
Ref: Hum Mol Genet. 2018 Aug 15; 27(16): 2805–2816. doi: 10.1093/hmg/ddy189

Rabbit anti-GAPDH (WB, 1:1000, overnight 4ºC/ Labned, LN2100751)
https://labned.com/gapdh-rabbit-unlb-antibody-ln2100751

Eukaryotic cell lines

Policy information about cell lines

Cell line source(s)	Oviductal and OSE organoid lines were established in Hubrecht Institute (Uppsalalaan 8, 3584 CT, Netherlands) from oviductal and OSE tissues of wild-type C57BL/6 or Cas9-EGFP (JAX stock #026175) reporter mice, respectively.
Authentication	No organoid line was authenticated.
Mycoplasma contamination	All cell lines tested negative for mycoplasma contamination.
Commonly misidentified lines (See ICLAC register)	No commonly misidentified cell lines were used in this study.

Animals and other organisms

Policy information about studies involving animals; ARRIVE guidelines recommended for reporting animal research

Laboratory animals	For mice experiments, 63 specific-pathogen-free (SPF) mice, NOD SCID gamma (NSG) strain, female, 8-12 weeks old mice were used in the study. The mice were kept in a constant temperature environment of 21°C (40–60% humidity) with a natural day/night light cycle in a conventional animal colony with free access to food and water. All the mice were housed in a pathogen-free vivarium in sterile, disposable microisolator cages and fed a sterile, irradiated diet with free access to sterile, irradiated water.
Wild animals	The study did not involve wild animals.
Field-collected samples	The study did not involve samples collected from the field.
Ethics oversight	Transplantation experiments were performed after institutional review by the Animal Ethics Committee of the Royal Netherlands Academy of Arts and Sciences (KNAW) with project license of AVD8010020151 and research protocol HI17.1001.

Note that full information on the approval of the study protocol must also be provided in the manuscript.

Human research participants

Policy information about studies involving human research participants

Population characteristics	Ovarian cancer tissues were obtained from consenting patients who underwent tumor resection. From the biobanked samples (samples thoroughly characterized under doi: 10.1038/s41591-019-0422-6), the tumor tissue was used from two following patients that were diagnosed with distinct types of benign ovarian tumors:<br>1) Primary cancer tissue from a 58-year-old patient (patient number E15-01841) diagnosed with mucinous borderline tumor

	(FIGO IC) located in left adnex with no previous neo-adjuvant chemotherapy.<br>2) Primary cancer tissue from a 57-year old patient (patient number E17-00555) diagnosed with serous borderline cystadenoma (FIGO IIIC) located in right ovary with no previous neo-adjuvant chemotherapy.<br><br>The aforementioned two patients' tissues were used in this manuscript to showcase the type-specific histological characteristics of the human benign tumors.
Recruitment	Relevant patients (diagnosed with ovarian cancer) were approached and included to the study when they agreed to take part in this study and signed informed consent. No self selection bias is anticipated.
Ethics oversight	The collection of patient data and tissue has been performed according to the guidelines of the European Network of Research Ethics Committees (EUREC) following European, national, and local law. The medical ethical committee UMC Utrecht (METC UMCU) approved the biobanking protocol: 14-472 HUB-OVI.

Note that full information on the approval of the study protocol must also be provided in the manuscript.

Flow Cytometry

Plots

Confirm that:

- [x] The axis labels state the marker and fluorochrome used (e.g. CD4-FITC).
- [x] The axis scales are clearly visible. Include numbers along axes only for bottom left plot of group (a 'group' is an analysis of identical markers).
- [x] All plots are contour plots with outliers or pseudocolor plots.
- [x] A numerical value for number of cells or percentage (with statistics) is provided.

Methodology

Sample preparation	Organoids were collected and dissociated into single-cell suspension via trypsinization. The cells were stained with Annexin V Apoptosis Detection Kit (88-8007-72, eBioscience) according to the manufacturer's instructions.
Instrument	A BD FACSCanto II system was used to analyse the samples
Software	Data was collected using BD FACSCanto II and analysed from BD FACSCanto II workstation.
Cell population abundance	No sorting.
Gating strategy	The cells were first gated for forward- and side-scatter area (FSC-A vs SSC-A) to select the cell population of interest and exclude the debris. Next, a sequential gating was performed to obtain single cells. The cells were first gated for forward-scatter area and height (FSC-A vs FSC-H) followed by gating for side-scatter area and height (SSC-A vs SSC-H), which allows for higher sensitivity in doublet exclusion. No stain, “PI only” and “Annexin V only” samples were used to set up the gates for the assay. Subsequently, PI and Annexin V-APC double-stained clones were analysed for apoptotic events and different population percentages recorded.

- [x] Tick this box to confirm that a figure exemplifying the gating strategy is provided in the Supplementary Information.
